# Molecular Insights of 7‑Azaindole Drugs and Their Intercalation in the Confined Space of Montmorillonite

**DOI:** 10.1021/acsomega.5c06055

**Published:** 2025-12-03

**Authors:** Ana Borrego-Sánchez, Eva M. García-Frutos, Margarita Darder, C. Ignacio Sainz-Díaz

**Affiliations:** † Department of Pharmacy and Pharmaceutical Technology and Parasitology, University of Valencia, Avda. Vicente Andrés Estelles SN 46100 Burjassot, Valencia, Spain; ‡ 69570 Instituto de Ciencia de Materiales de Madrid (ICMM, CSIC) Campus de Cantoblanco, Madrid 28049, Spain; § 16379 Instituto Andaluz de Ciencias de la Tierra (Consejo Superior de Investigaciones Científicas). Av. de las Palmeras 4 18100 Armilla, Granada, Spain

## Abstract

Two derivatives of the group of 7-azaindoles, 1-benzyl-3-(piperidin-1-ylmethyl)-1H-pyrrolo [2,3-*b*] pyridine, and 1-benzyl-5-methoxy-3-(piperidin-1-ylmethyl)-1H-pyrrolo [2,3-*b*] pyridine as mono-oxalate salts are studied in this work as potential neuroprotective drugs for the treatment of Alzheimer’s disease. Previously, we studied the use of a natural montmorillonite clay mineral as a candidate for a drug delivery system, finding that these drugs can be intercalated into the confined interlayer space of montmorillonite and subsequently released in a human medium for therapeutic use. However, some aspects of this study could not be explained. This work has studied this process at the atomic and molecular levels by using the Interface force field (FF). Initially, this methodology was validated in this work, reproducing the experimental crystal structure of these 7-azaindole drugs. Then, this FF was applied to calculate the intercalation of these drugs by cation exchange into montmorillonite according to the experimental results. Our calculations have reproduced this intercalation at the cation exchange capacity at the molecular level, finding that the experimental structure can only be justified with the intercalation of five drug molecules per 4 × 2 × 1 supercell of clay mineral inside the confined interlayer space. In addition, this intercalation does not produce a monolayer disposition postulated initially from experiments. On the contrary, our molecular dynamics simulations show that the intercalated molecules adopt a disordered disposition with a certain tendency to form a bilayer configuration in the confined interlayer space of montmorillonite. Besides, the spectroscopic infrared properties are useful for monitoring the preparation and encapsulation processes of pharmaceutical drugs. Then, these properties were studied experimentally and calculated theoretically. The calculated frequencies of the crystal structure of these 7-azaindole drugs allowed assignments of the experimental FT-IR spectra. This collaborative work with experimental and theoretical research enhances the knowledge for a promising drug delivery system for anti-Alzheimer therapy.

## Graphical abstract

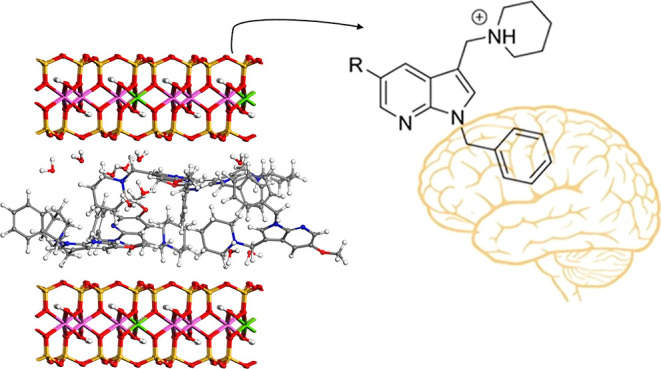

## 1 Introduction

The search for new drugs for the treatment of Alzheimer’s disease (AD) and other dementias represents a huge challenge, as there were an estimated 55 million people affected by these diseases worldwide in 2020 and the number is expected to rise to 78 million by 2030.^1^ AD is a multifactorial neurodegenerative disease in which many pathological processes are triggered, causing neuronal damage, including the formation of neurofibrillary tangles due to the hyperphosphorylation of Tau protein or the formation of extracellular amyloid plaques, among others.^2–5^ Although there is still no known cure for AD, several drugs are available to manage its symptoms, and research is underway to find new drugs that can slow or even stop the disease. Given the multifactorial etiology of AD, multitarget-designed ligands (MTDLs) appear as a promising strategy for its treatment. Following this MTDL approach, a series of gramine-based neuroprotective drugs (7-azaindole derivatives) showing the ability to modulate the hyperphosphorylation of Tau protein were developed in the past decades as promising neuroprotective drugs.^6–8^ Oral administration is a convenient route for the administration of drugs to AD patients, but it would be necessary to formulate a drug with prolonged drug release since AD is a chronic disease. The use of nanomaterials acting as nanocarriers of the drug molecules is commonly proposed to overcome this drawback, providing a controlled delivery of the drug and keeping its concentration in the bloodstream within the therapeutic range for a long period of time.^9^ Moreover, these gramine-based neuroprotective drugs are unstable in acidic environments such as the gastric tract,^7^ reducing their bioavailability and, in consequence, reducing the treatment efficacy. The clay minerals can also offer protection in harsh environments.

Clay minerals are extensively used in the pharmaceutical and cosmetic industries,^10,11^ and many research studies in the past decades have focused on their use as nanocarriers for a wide variety of drugs due to their exceptional features, such as nontoxicity, biocompatibility, sorption capacity, and ion-exchange properties.^12–15^ Because of their abundance in nature and suitable properties, the most widely used clays in drug delivery are smectites such as montmorillonite, kaolinite, and hectorite.^12,16–18^ Montmorillonite, a 2:1 aluminosilicate with a high cation exchange capacity around 70 to 130 mequiv/100 g,^19,20^ can easily intercalate neutral or positively charged organic molecules in its interlayer region. Thus, many formulations have been prepared by incorporating a wide variety of drug molecules in montmorillonite, such as metformin used for the treatment of type II diabetes,^21^ tamoxifen used in cancer treatment,^22^ praziquantel antiparasitic drug for schistosomiasis,^23^ donepezil used in the treatment of Alzheimer’s disease,^24^ or antibiotics such as amoxicillin,^25^ neomycin,^17^ or oxytetracycline^26^ used to treat bacterial infections, among many other examples.^16,27^ Similarly, new drugs synthesized for Alzheimer’s disease based on indole and aza-indole derivatives have also been intercalated in montmorillonite to improve the bioavailability of these drugs in oral administration.^8^

Computational molecular modeling of the drugs intercalated in montmorillonite is carried out to study the interaction established between the molecules and the inorganic support used as nanocarriers. These clay minerals provide an interesting interlayer space where molecules can be confined at the nanoscale level. However, the small crystal size and the high crystallographic disorder make it difficult to monitor and understand the intercalation process of these materials at the molecular level. Computational molecular modeling is rising as a useful tool for interpreting the experimental behavior related to physical–chemical properties^28,29^ and reactivity^30^ of these minerals. In the past decades, this methodology has become a promising tool for studying the intercalation of bioactive molecules^31–33^ for pharmaceutical applications.^34–36^

New drug delivery systems have been recently developed by intercalation of two neuroprotective drugs, 7-azaindole (7AI) and 5-methoxy-7-azaindole (5OMe-7AI)-derivative mono-oxalate salts (Figure 1), in montmorillonite, aiming at increasing their bioavailability in oral administration.^8^ In the current work, a theoretical study is carried out in order to determine the arrangement of both molecules in the interlayer space of montmorillonite as well as the intermolecular interactions of the different drugs and their interaction with the silicate layers, compared with experimental spectroscopic studies.

**Figure 1:**
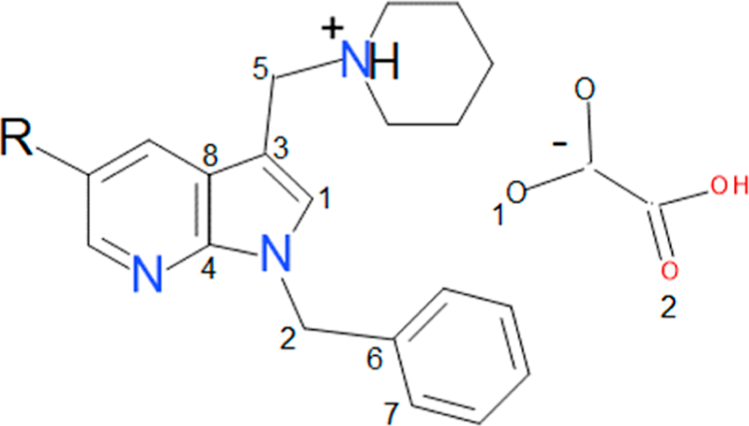
Chemical structure of the synthesized compounds 7AI (R=H) and 5OMe-7AI (*R* = OMe)-derivative mono-oxalate salts.

## 2 Materials and Methodology

### 2.1 Materials Synthesis and Characterization

The 7AI [1-benzyl-3-(piperidin-1-ylmethyl)-1H-pyrrolo [2,3-*b*] pyridine] and 5OMe-7AI [1-benzyl-5-methoxy-3-(piperidin-1-ylmethyl)-1H-pyrrolo [2,3-*b*] pyridine]-derivative mono-oxalate salts were synthesized, and their crystal structures were described by Lajarín-Cuesta et al.^7^ and García-Vázquez et al.^8^ The compounds have appropriate purity for pharmacological studies from elemental chemical analysis and high-resolution mass spectrometry. Their lack of cytotoxicity was confirmed in cell viability tests with SH-SY5Y neuroblastoma cells, measured by the method of MTT [3-(4,5-dimethylthiazol-2-yl)-2,5-diphenyltetrazolium bromide] reduction.^7,8^ The preparation of the hybrid compounds with 7-AI and 5OMe-7AI intercalated into the Na montmorillonite solid has been described elsewhere.^8^ In this work, a specific FT-IR spectroscopy study of these drugs and their intercalation products has been performed by using a BRUKER IFS 66 v/S spectrophotometer, recording the spectra from 4000 to 400 cm^–1^ with a 2 cm^–1^ resolution using KBr pellets.

### 2.2 Models

A model of montmorillonite was created in order to reproduce the experiments and to study at the atomistic level the intercalation of the drug in the interlayer space of this natural material. The clay mineral used in the experimental part was the homoionic Na-montmorillonite (MMT) with the ideal formula (Na)_0.33_(AlMg)_2_Si_4_O_10_(OH)_2_·nH_2_O and commercialized as CloisiteNa by Southern Clay Products.^8^ An atomic model of the MMT crystal structure was created with a chemical composition similar to that used experimentally. Specifically, a periodical crystal structure of MMT was designed, generating a 4 × 2 × 1 supercell with the formula Na_5_(Al_27_Mg_5_)­Si_64_O_160_(OH)_32_·10H_2_O (325 atoms). The Mg^2+^ cations were placed taking into account a maximum dispersion along the octahedral sheet according to previous works.^28,29^ Our experimental TGA of these samples showed a water content of 3%. Taking into account the chemical composition of this clay, one unit cell weighs 740 g/unit-cell, and then a 4 × 2 × 1 supercell weighs 5920 g/supercell. Considering 10 water molecules (180 g), the total weight will be 6100 g/supercell-wet, that is, 180/6100 = 0.0285 = 3% of water. Hence, ten molecules of water per supercell were included around sodium cations in the interlayer space of the clay.

Subsequently, on this clay model, the intercalation of two 7-azaindole derivative molecules was studied. Specifically, these drugs were described and called compounds 7AI and 5OMe-7AI mono-oxalate salts by García-Vázquez et al.^8^ (Figure 1). The crystal structures of these drugs were taken from the Cambridge Crystallographic Data Centre with ref CCDC-1936481 for the mono-oxalate salt of 7AI, and CCDC-1936482 for the mono-oxalate salt of 5OMe-7AI monosolvated. Both structures were transformed to P1 symmetry and were used for our calculations. For further intercalation studies, each molecule was extracted from the above crystal structure as a cation and also as the mono-oxalate salt.

For the intercalation complex models, the interlayer space is expanded to higher *d*(001) values, and each organic molecule was placed in the center of the interlayer space, avoiding too close interatomic distances with the water molecules, Na^+^ cations, and mineral surface. These 7-azaindoles are monovalent cations. Thus, the intercalation of one molecule of the drug was performed by cation exchange, substituting each one per one interlayer sodium cation for maintaining the whole system electrically neutral after the intercalation. According to experimental information, when the cation exchange of the clay was complete, all sodium cations were substituted by the 7-azaindol molecules to reproduce the intercalation experiments of the drug, completing the cation exchange capacity.

### 2.3 Computational Methodology

Atomistic calculations using force fields (FFs) based on empirical interatomic potentials were performed for optimizing the molecular and crystal structures of the drugs, the MMT crystal structure, and the intercalation complexes, applying periodic boundary conditions. The use of FFs has been successfully applied in the study of clay minerals, such as Interface,^37^ CLAYFF,^38,39^ and others,^40^ for crystallographic,^41^ water swelling,^42^ and absorption properties.^37^ Specifically, the Interface_v1.5 FF^37^ is used in this work because previously this FF described well the phyllosilicate structure with water and organic molecules.^35^ The energy minimizations and the geometrical optimizations were performed with the Forcite program.^43^ For nonbonding interactions, the Coulomb and van der Waals interactions were calculated with the Ewald method, applying a cutoff of 15 Å. The van der Waals (vdW) interactions were calculated with the Lennard-Jones potential, V­(r) = ϵ­[(σ/r)^12^ – 2­(σ/r)^6^, within this FF. The net atomic charges of the clay and drug molecules were set manually, considering the FF used. The SPC water molecules’ charges were used by applying charge values of +0.41 and −0.82 for H and O atoms, respectively.^44,45^

In order to validate the FF used, the 7AI and 5OMe-7AI molecules were also optimized using DFT calculations with the DMol^3^ program. Specifically, we used the generalized gradient approximation (GGA) with the revised Perdew–Burke–Ernzerhof exchange–correlation functional (RPBE).^46^ DFT semicore pseudopotentials (DSPP) with the Double Numerical plus Polarization (DNP) basis set were selected, and the convergence threshold for the self-consistent field (SCF) was 10^–6^ Ha. The net atomic ESP charges (charges associated with the electrostatic potential) were calculated.^47^ These charges were useful for applying in previous calculations with the FF.^34,48^ Nevertheless, two comparative strategies were tested: (a) to optimize the structure with Interface and with net atomic charges of their own FF and (b) to optimize the structure previously with DFT and later to reoptimize the structure with the FF applying the ESP charges.

The infrared spectral frequencies were calculated after fully optimizing the geometries with Interface. The Hessian matrix (second derivative of energy with respect to atomic displacements) was then obtained by using the harmonic approach. The absence of negative frequency values confirmed that the system is in a minimum energy state on the potential energy surface. The frequencies of the main vibration modes were calculated from this Hessian matrix.^43^

On the other hand, the drug–clay intercalation complexes were generated using a Metropolis Monte Carlo method with Interface. This methodology was used for exploring randomly different conformations and orientations in the intercalation of five molecules of each drug compound in the interlayer space of the MMT with 10 water molecules per MMT supercell. Both adsorbate molecules were optimized previously under the same conditions separately. A simulated annealing was applied with 10 temperature cycles of 100,000 Monte Carlo steps per cycle, where different configurations of the adsorbates with respect to the surface were searched, avoiding local minima, including optimization of geometry with a convergence tolerance of 2 × 10^–5^ kcal/mol and 0.001 kcal mol^–1^Å^–1^. Only the positions and orientations of the adsorbate molecules were sampled. The adsorbate molecules were treated as rigid bodies with a low degree of torsional flexibility, and any internal degrees of freedom that the adsorbate components may possess on the substrate surface were ignored. The most stable complex was selected and fully reoptimized at variable volumes with the FF and following the same methodology described above.

Molecular dynamics simulations of these intercalated complexes were performed with 1 fs steps during 5 ps within the *NVT* ensemble with the NOSE thermostat at 298 K and the same above FF.

## 3 Results and Discussion

### 3.1 Molecular and Crystalline Structures of the (7AI) and (5OMe-7AI) Mono-oxalate Salts

The molecular structure of the 7AI (a) and 5OMe-7AI (b) oxalate salt drugs extracted from the crystal data was optimized under vacuum with Interface (Figure 2).

**Figure 2:**
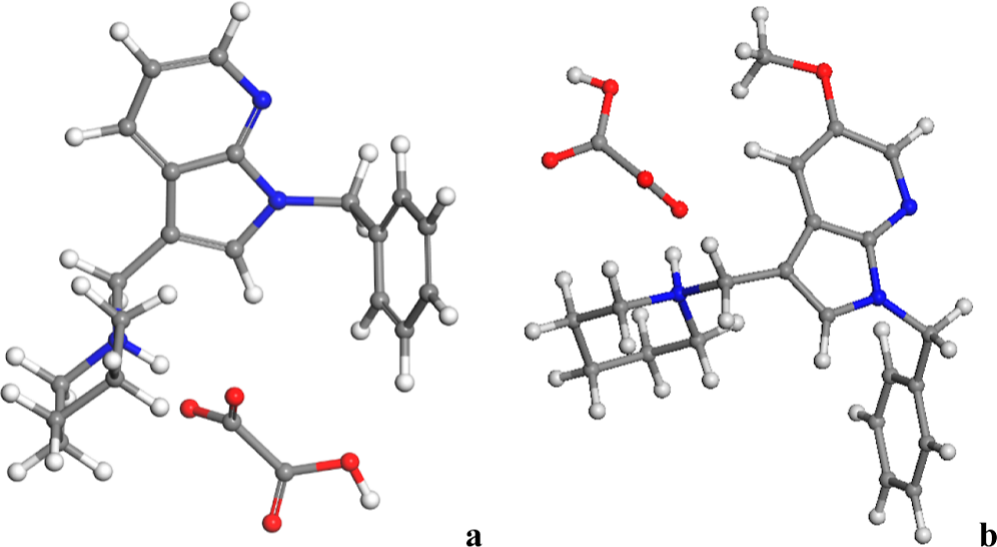
Molecular structure of 7AI (a) and 5OMe-7AI (b) derivative mono-oxalate salts optimized as isolated molecules with Interface. The carbon, hydrogen, nitrogen, and oxygen atoms are described in gray, white, blue, and red colors, respectively. This criterion is extended to the rest of the figures of this work.

In Table 1, the main geometrical features of the optimized molecules in the gas phase were recollected, comparing with our experimental values from the solid state. The results show that, using atomic charges calculated with Interface, the geometrical features were in general closer to the experimental ones (lower RMSD values) compared with the other methodology tested. In both compounds, the piperidine and aromatic benzyl rings are twisted quasi-perpendicular with respect to the indole ring, with dihedral angles within the 100—122° range. In general, the bonds of the N or C atoms of the aromatic ring with C atoms are better described by Interface. The bonds between C and O atoms were also well described with Interface, as well as the O–H bonds. Nevertheless, the differences are small. Hence, we can consider this FF with its own atomic charges as a valid method for describing these 7AI and 5OMe-7AI mono-oxalate salts (Table 1).

**Table 1:** Main Geometrical Features (Bond lengths in Å and Angles in °) of the Optimized Molecular Structure of the 7AI and 5OMe-7AI Mono-oxalate Salt Derivatives and Experimental Data

features^a^	7AI^b^	7AI^c^	5OMe-7AI^b^	5OMe-7AI^c^
N_5‑ring_-CH_2_ (N–C2)	1.482 (1.488)	1.454(2)	1.484 (1.486)	1.473(7)
N_5‑ring_-CH (N–C1)	1.388 (1.386)	1.369(2)	1.390 (1.358)	1.354(7)
N_6‑ring_-C (N–C4)	1.312 (1.307)	1.334(2)	1.310 (1.312)	1.323(7)
C_5‑ring_-CH_2_ (C3–C5)	1.494 (1.480)	1.490(2)	1.493 (1.491)	1.482(8)
N_pipe_-H	1.027 (1.019)	0.934(17)	1.013 (1.025)	0.90
N_pipe_-CH_2_ (N–C5)	1.526 (1.526)	1.504(2)	1.529 (1.527)	1.518(7)
C=O^d^(O1)	1.244 (1.247)	1.2321(17)	1.245 (1.244)	1.236(6)
C=O^d^(O2)	1.209 (1.209)	1.1962(18)	1.211 (1.208)	1.195(6)
C–OH	1.371 (1.379)	1.2972(17)	1.367 (1.368)	1.291(6)
O–H	0.950 (0.953)	0.92	0.949 (0.950)	0.92
C=O···HN	1.734 (1.660)	1.859(17)	1.714 (1.825)	2.02(5)
C–OCH_3_			1.387 (1.386)	1.358(6)
O–CH_3_			1.425 (1.426)	1.412(7)
RMSD	0.050 (0.072)		0.069 (0.094)	
CC_benz_-C_(H2)_–N_5‑ring_ (C7–C6–C2–N)	113.0 (113.3)	114.51(17)	113.4 (122.5)	117.2(6)
C–C_5‑ring_-C_(H2)_–N_(H)pipe_ (C8–C3–C5–N)	116.4 (114.9)	114.99(12)	100.0 (100.0)	116.5(5)

a References to Figure 1 are in brackets; N_5‑ring_ means the N atom of the small cycle of the indol moiety; N_6‑ring_ is the N atom of the large ring of indol; N_pipe_ is the N atom of the piperidine ring; C_benz_ are the C atoms of the benzene ring.

b Calculated with Interface and with net atomic charges of their own FF (values optimized with DFT, then with Interface by applying ESP atomic charges in brackets).

c Experimental values, errors in brackets.^8^

d From the oxalate anion, O1 and O2 are from the carboxylate and carboxylic groups, respectively.

The crystal structure of the 7AI and 5OMe-7AI mono-oxalate salts was fully optimized (atomic positions and lattice cell parameters) with Interface with the atomic charges of their own FF (Figure 3).

**Figure 3:**
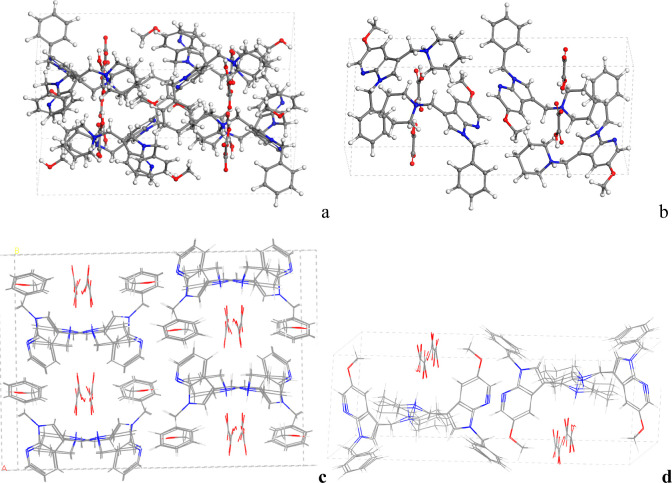
Optimized crystalline structure of the 7AI (a) and 5OMe-7AI (b) derivative mono-oxalate salts. Superposition of the experimental and calculated crystal structures of 7AI (c) and 5OMe-7AI (d).

In Table 2, the values of lattice parameters and intermolecular distances of the mono-oxalate salt crystal optimized at variable volume were compared with the experimental data of the crystal. The deviation between experimental and calculated cell parameters was lower than 5%, being larger in intermolecular H bonding. The superposition of the experimental and calculated crystal structures of both drugs indicates good agreement. The results corroborate that the Interface FF reproduces well the structures of both drugs. Hence, this FF can be considered valid for its application in the study of clay–drug complexes in this work. The unit cell of the 7AI crystal is formed by 8 aza-indole molecules with their corresponding semioxalate counterions and 4 methanol molecules. On the other hand, the unit cell of the 5OMe-7AI crystal is formed by 4 aza-indole molecules with their four semioxalate counterions. In both structures, the oxalate groups form chains with intermolecular hydrogen bonds of the NH group of the piperidine moieties.

**Table 2:** Values of Lattice Cell Parameters and Main Intermolecular Distances of the Crystal Structures of the 7AI and 5OMe-7AI Mono-oxalate Salts Fully Optimized at Variable Volume and Compared with the Experimental Lattice (Distances in Å and Angles in °)

lattice parameters	7AI^a^	7AI^b^	5OMe-7AI^a^	5OMe-7AI^b^
a	11.797 (0.05)	11.2063(3)	8.225 (0.05)	8.7101(12)
b	16.237 (0.04)	16.8396(6)	23.931 (0.03)	23.329(4)
c	22.872 (0.01)	23.0117(8)	11.683 (0.05)	11.1268(16)
α	90.0	90.0	90.0	90.0
β	90.0	90.0	110.5 (0.003)	110.219(8)
γ	90.0	90.0	90.0	90.0
NH_aza_···OC	1.859	1.859(17)	1.808 (0.10)	2.02(5)
NH_aza_···OH	2.130 (0.12)	2.432(16)		2.27(5)
N_aza_···HO	1.680 (0.18)	2.043(2)		

a Calculated with the Interface FF; the relative deviations are in brackets.

b Experimental data, errors in brackets.^8^

### 3.2 Intercalation Modeling in Montmorillonite

These compounds have low solubility in water, but they could be completely dissolved in water/methanol mixtures, allowing their intercalation in the layered clay as shown by García-Vazquez et al.^8^ In fact, in the case of hydrophobic drugs like these, the interaction with clay minerals can facilitate their solubility, as found in some of our previous studies.^23,49^ However, these experiments cannot give us information related to the distribution of these intercalated molecules in the confined interlayer space. Hence, an atomistic modeling approach is used to describe this intercalation at the atomic and molecular levels in this work. Taking into account the validation of the Interface FF for reproducing the crystal structure of these drugs described above, we use this FF for studying the intercalation of these drugs into the confined interlayer space of MMT.

Following previous experimental data,^8^ the crystal structure of MMT has the ideal formula (Na)_0.66_(AlMg)_4_(Si_8_)­O_20_(OH)_4_·nH_2_O per unit cell. Then, for intercalation studies, a 4 × 2 × 1 supercell with ten molecules of water was generated and optimized using Interface. This MMT with water, Na_5_(Al_27_Mg_5_)­Si_64_O_160_(OH)_32_·10H_2_O, was optimized at variable volume, obtaining a basal spacing of *d*(001) = 11.5 Å (Figure 4a). The water H atoms were oriented to the basal O atoms of the clay mineral surface by electrostatic forces, as was expected. The water O atoms were oriented to the Na^+^ cations by electrostatic interactions (Figure 4a). One molecule of 7AI was exchanged with a Na^+^ cation and was placed as a cation in the center of the interlayer space of MMT. After optimization, the drug remains close to the center of the interlayer, adopting an orientation coplanar with the plane 001 of the mineral surface in a more extended configuration than out of the interlayer (Figure 4b). Some water O atoms are oriented to the positively charged N atoms of the polar zone of the drug cation. However, the hydrophobic zone of the drug has pushed away the other water molecules, which have a disordered geometry. Some Na^+^ cations are also coordinated by the basal O atoms of the mineral surface along with the water O atoms (Figure 4b).

**Figure 4:**
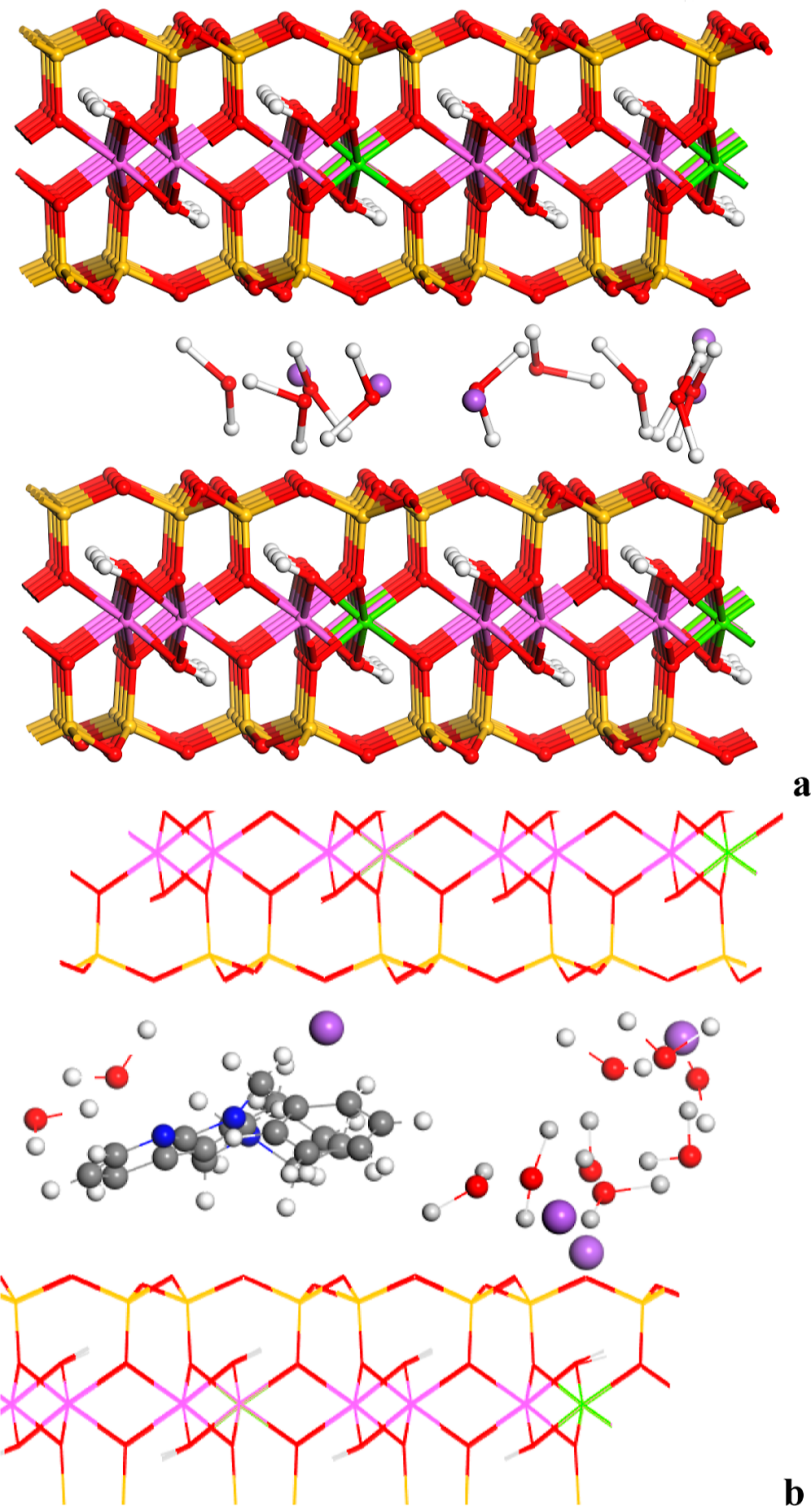
Crystal structures of the 4 × 2 × 1 supercell of MMT (a) and with one 7-AI molecule intercalated by cation exchange (b), optimized with Interface. The Si, Al, Mg, and Na atoms are described in yellow, pink, green, and violet colors, respectively. This criterion is extended to the rest of the figures of this work.

Experimentally, the specific number of drug molecules adsorbed in the interlayer space of clay or on the external surface of the mineral is not possible to know. However, previous experimental data indicated that the sodium cation exchange by drug molecules was practically completed.^8^ Theoretical calculations will allow corroborating whether the entire cationic exchange of sodium by 7-azaindole molecules corresponds to the spacing obtained by X-ray diffraction (XRD) experimentally.

In our previous work,^8^ the adsorbed amount of drug was quantified, 82–87 mequiv./100 g clay. Taking into account the chemical composition of this clay, one unit cell weighs 740 g/unit-cell, that is, 5920 g/4 × 2 × 1 supercell. Considering 10 water molecules per supercell (180 g), the total weight will be 6100 g/4 × 2 × 1 supercell-wet. Hence, the amount of drug equivalent per supercell will be (6100 × 82/100)/1000 = 5.0–5.3 molecules per 4 × 2 × 1 supercell. Therefore, two hybrid complexes were modeled and optimized to study the intercalation of five molecular cations of 7AI and 5OMe-7AI exchanged by the five sodium cations of the montmorillonite supercell with 10 water molecules. To do this, a Monte Carlo method with Interface was used in order to randomly explore different conformations and orientations in the intercalation of the five drug molecules in the interlayer space of the MMT supercell in each complex. The initial interlayer spacing *d*(001) of MMT was 22 Å. This value was created to provide enough space to arrange all absorbate molecules before the optimization. Subsequently, using Monte Carlo-simulated annealing methods, the water molecules and the 5 drug molecules were adsorbed in the interlayer space of the clay, obtaining different orientations and conformations between MMT, the drug, and water molecules. After this calculation, the most stable complex was selected and subsequently fully optimized at a variable volume. The resultant intercalation complexes with five drug molecules fully optimized at variable volume yield a *d*(001) spacing of 19.4 and 19.2 Å for MMT­(7AI)_5_ and MMT­(5OMe-7AI)_5_, respectively (Figure 5). These values reproduce approximately the *d*(001) spacing obtained in the above experiments of this work, although the calculated values are slightly lower than the experimental values.^8^ This can be due to the amount of water in the interlayer space. We optimized the same models with 4 times the water proportion, and the *d*(001) spacing increased to 19.6 and 19.5 Å for MMT­(7AI)_5_ and MMT­(5OMe-7AI)_5_, respectively. After reaching the maximum cation exchange capacity of MMT, the *d*(001) spacing of the MMT­(7AI)_5_ complex is slightly higher than that of the MMT­(5OMe-7AI)_5_ one. The same behavior was observed experimentally, 20.1 and 19.1 Å for MMT­(7AI)_5_ and MMT­(5OMe-7AI)_5_, respectively.^8^

**Figure 5:**
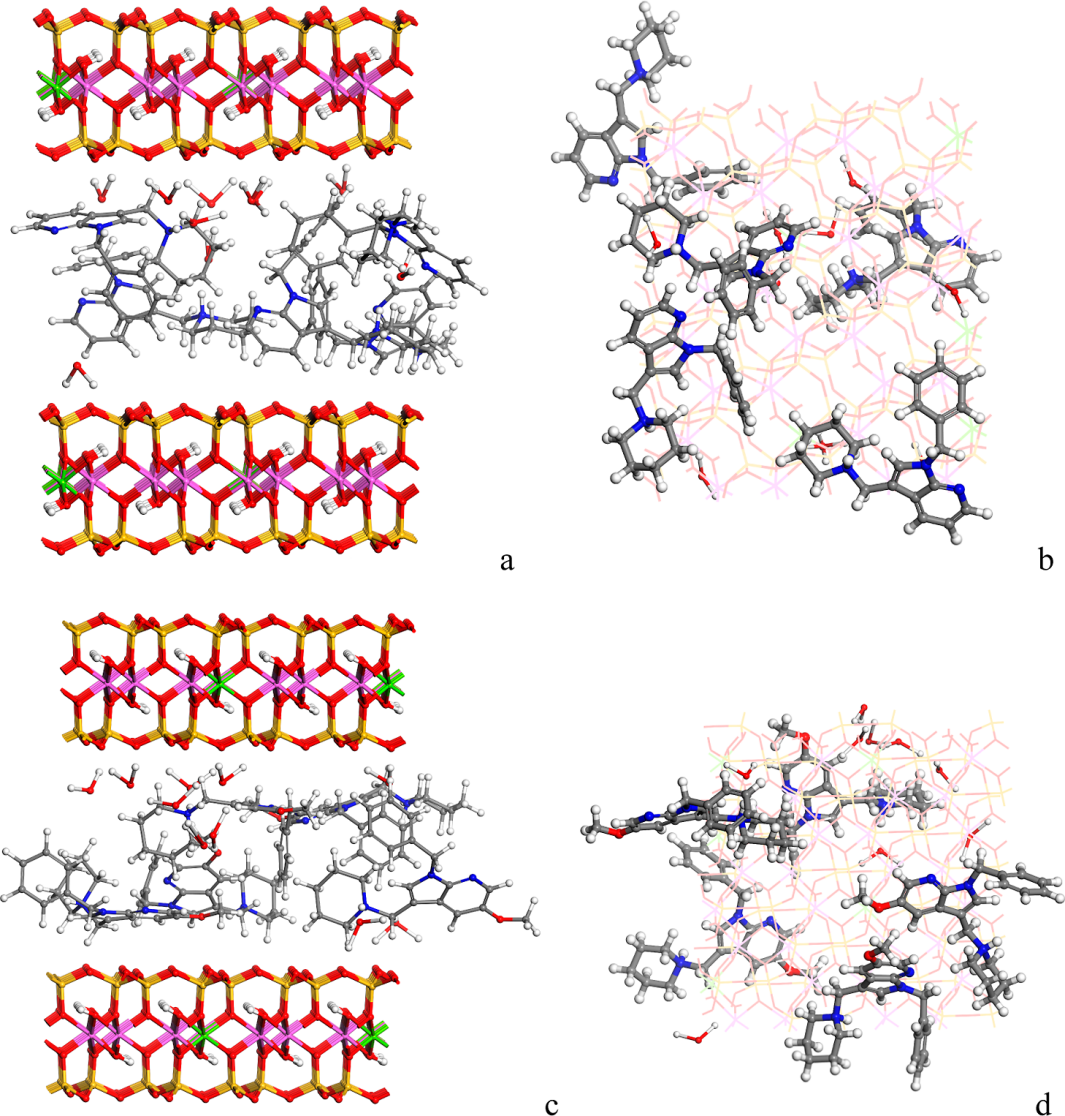
Optimized intercalation complexes of MMT­(7AI)_5_ [(a) viewed from the (010) plane and (b) viewed from the (001) plane] and MMT­(5OMe-7AI)_5_ [(c) viewed from (010) plane and (d) viewed from the (001) plane] intercalated in the confined interlayer space of MMT after reaching the maximum cation exchange capacity (five molecular cations of drug per 4 × 2 × 1 supercell).

After optimization of the hybrid complex, the 7-azaindole molecules showed electrostatic interactions with the surface of montmorillonite. Specifically, interactions between the drug H atoms and the basal tetrahedral O atoms of the clay surface were found. The H atoms of the CH_2_ and NH groups of the piperidine moiety interact with the clay surface O atoms, *d*(H···O_MMT_) = 2.31 Å and 2.67 Å, respectively. Also, electrostatic interactions were found between the H atoms of the CH_2_ group and the CH of the aromatic rings of the drug with the O atoms of the clay with *d*(H_A_···O_MMT_) = 2.56 Å and 2.31 Å, respectively. Moreover, strong hydrogen bonds between water H atoms and the clay surface O atoms with *d*(H_w_···O_MMT_) = 1.47 Å were observed. With the structures extracted from the molecular dynamics simulations, we can observe that the disposition of these molecules into the interlayer space is disordered with a certain bilayer configuration (Figure 6). Two maxima are observed in the concentration profile of C atoms along the 001 direction of the interlayer space in both complexes (Figure 6a,b). The same conclusion can be found from the concentration profile of N atoms (Figure 6c,d). In this last profile, two double peaks are observed for MMT­(7AI)_5_ due to the N atoms at indol and piperidine rings that are in different planes. This is not observed in MMT­(5OMe-7AI)_5_.

**Figure 6:**
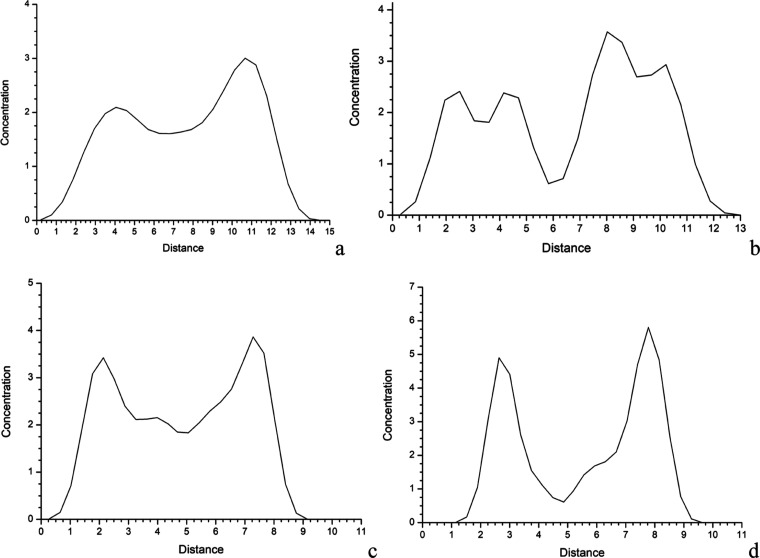
Concentration profile along the (001) interlayer projection of C (a) and N (b) atoms of MMT­(7AI)_5_ and C (c) and N (d) atoms of MMT­(5OMe-7AI)_5_ for the frames sampled from the molecular dynamics simulations.

We can summarize that the above models indicate the maximum amount of 7-azaindoles intercalated in the interlayer of montmorillonite by exchange cations and corroborate the interlayer *d*(001) spacing observed experimentally.^8^

#### 3.2.1 Intercalation Energy

The intercalation of 7-azaindole drug molecules in the interlayer space of MMT follows a cation exchange process. In this process, we consider two main interactions: the interactions of the cation with the mineral surface and with water. To calculate the intercalation energy (by cation exchange), the cation–water interactions should be the same in the complete process. This has been simulated by creating models with the cations in the water, following previous works.^35^ For that, periodical boxes with 10 molecules of water and Na^+^ or AZA^+^ neutralized with an anion (mono-oxalate) were created, maintaining similar dimensions as in the clay interlayer space. So, both cations are hydrated in the media or in the interlayer space with 10 water molecules. Moreover, the structure of montmorillonite with 10 water molecules and five sodium cations was also generated. Each system was optimized with an Interface.

Then, the intercalation energy can be calculated by a cation exchange reaction such as (1)

1 $${MMT}^{.}{\left({Na}^{+}\right)}_{5}+5{AZA}^{+}{Salt}^{-}{\;↔\;MMT}^{.}{\left({AZA}^{+}\right)}_{5}+{5Na}^{+}{Salt}^{-}$$

where MMT­(Na^+^)_5_ means the initial MMT supercell crystal structure with the hydrated Na^+^ cations, AZA^+^Salt^–^ is the box of 7AI or 5OMe-7AI salt hydrated with water molecules, MMT­(AZA^+^)_5_ is the clay with the 7AI or 5OMe-7AI cationic drugs intercalated, and Na^+^Salt^–^ is the box with Na^+^ salt hydrated with water molecules.

Taking into account this reaction, the exchange energy, Δ*E*ex, is calculated following eq 2

2 $$\Delta Eex=E\left[MMT{\left({AZA}^{+}\right)}_{5}\right]+5E\left({Na}^{+}{Salt}^{-}\right)-E\left[MMT{\left({Na}^{+}\right)}_{5}\right]-5E\left({AZA}^{+}{Salt}^{-}\right)$$

MMT­(Na^+^)_5_ will have the supercell of MMT, 5 Na cations, and 10 water molecules per supercell; AZA^+^Salt^–^ is the hydration box of one drug cation neutralized with the salt anion and surrounded by 10 water molecules; MMT­(AZA^+^)_5_ is the supercell of MMT with 10 water molecules and five AZA^+^ cations; and Na^+^Salt^–^ is the hydration box of one Na^+^ cation neutralized with a Salt^–^ anion and 10 water molecules.

The calculated intercalation energies were −2.26 kcal/mol (−0.452 kcal/mol per molecule) and −7.01 kcal/mol (−1.402 kcal/mol per molecule) for the complexes with 7AI and 5OMe-7AI drugs, respectively. Both indicate favorable intercalation of the aza cations in the interlayer space of montmorillonite. The difference in the intercalation energies between the two adsorbates is very small because the molecular structures are similar. These energy differences are at the same level as those between different configurations of the same molecule in the interlayer space. Nevertheless, the presence of the methoxy group could justify the slightly higher value in 5OMe-7AI. This is observed experimentally, where the *d*(001) spacing with 5OMe-7AI is smaller than with 7AI,^8^ due to a higher interaction with the mineral surface of the interlayer space, justifying its slightly higher intercalation energy found theoretically. The main interactions in the systems with the intercalated drugs are nonbonding forces, electrostatic and van der Waals interactions. The results corroborate the total cation exchange observed experimentally between the clay and drugs.

The cell parameters and the atomic coordinates of these structures are available in the Supporting Information section.

### 3.3 Spectroscopical Properties

Infrared spectroscopy shows the vibration modes of the bonds between atoms forming the molecules. At the same time, we study these structures at the atomistic level. Hence, this spectroscopic technique can be a good bridge between experimental and theoretical approaches in our research. The frequencies of the main normal vibration modes of 7AI and its intercalated models were calculated. The crystal structure of the 7AI drug was chosen for this spectroscopic analysis because the experimental data come from this drug in the solid state in a crystal form. This approach is more realistic than calculations on isolated molecules as a classical way observed in many previous theoretical works.

The experimental spectra do not allow easy detection of the frequency variations of the drug molecules during the intercalation process due to their low resolution (Figure 7). Then, our calculated frequencies of the 7AI crystal structure (Table 3) are similar to those of the experimental data. So, these calculations allow us to assign the experimental bands observed in the infrared spectra. The calculated bands of the ν­(OH) mode can distinguish the frequencies of the mono-oxalate OH groups and the methanol OH groups. The last one appears at lower frequencies. The experimental band is complex and wide; we can assign the higher-frequency zone of this band to the mono-oxalate anion, and the lower-frequency zone can be assigned to the methanol OH groups. The ν­(NH) band appears at a lower frequency, 3273–3271 cm^–1^, than other amino groups due to the hydrogen bonds between the NH H atom and the mono-oxalate O atoms. The ν­(CH) bands are complex for assignment and can be observed as a unique band with a superstructure because they have different electronic properties. Nevertheless, our calculations can distinguish the vibration modes of each C–H bond of this indole ring. The C–H bond, which is in the position para with respect to the pyridine N atom of the indole moiety, shows the highest ν­(CH) frequency, and its ν­(C–H) vibration mode can be assigned to the lower frequency shoulder of the experimental band observed at 3300—3200 cm^–1^ overlapping with the ν­(NH) band. The ν­(CH) mode of the CH group at the position ortho with respect to the pyridine N atom of the indole moiety appears at a lower frequency than the former one and at a similar frequency to the CH group of the small 5-member indole ring. The CH group in the position meta with respect to the N atom of the indole moiety shows the lower ν­(CH) frequency of the indole ring. The CH bonds of the aromatic benzenic ring show lower frequencies than those of the above CH groups. Our calculations can distinguish each CH group of this benzenic ring. The CH bond in the position meta with respect to the backbone substituent appears at a higher frequency, 3104–3092 cm^–1^, than the rest of aromatic CH groups, overlapping with the ν­(CH) of the indole moiety. The ν­(CH) modes of the piperidine ring appear at lower frequencies, 3017–2915 cm^–1^. In the same range appear the ν­(CH) bands of the backbone CH_2_ groups and the methyl group of the methanol molecule embedded in the crystal structure. All of these last groups can be grouped in the experimental band at 2989–2862 cm^–1^.

**Figure 7:**
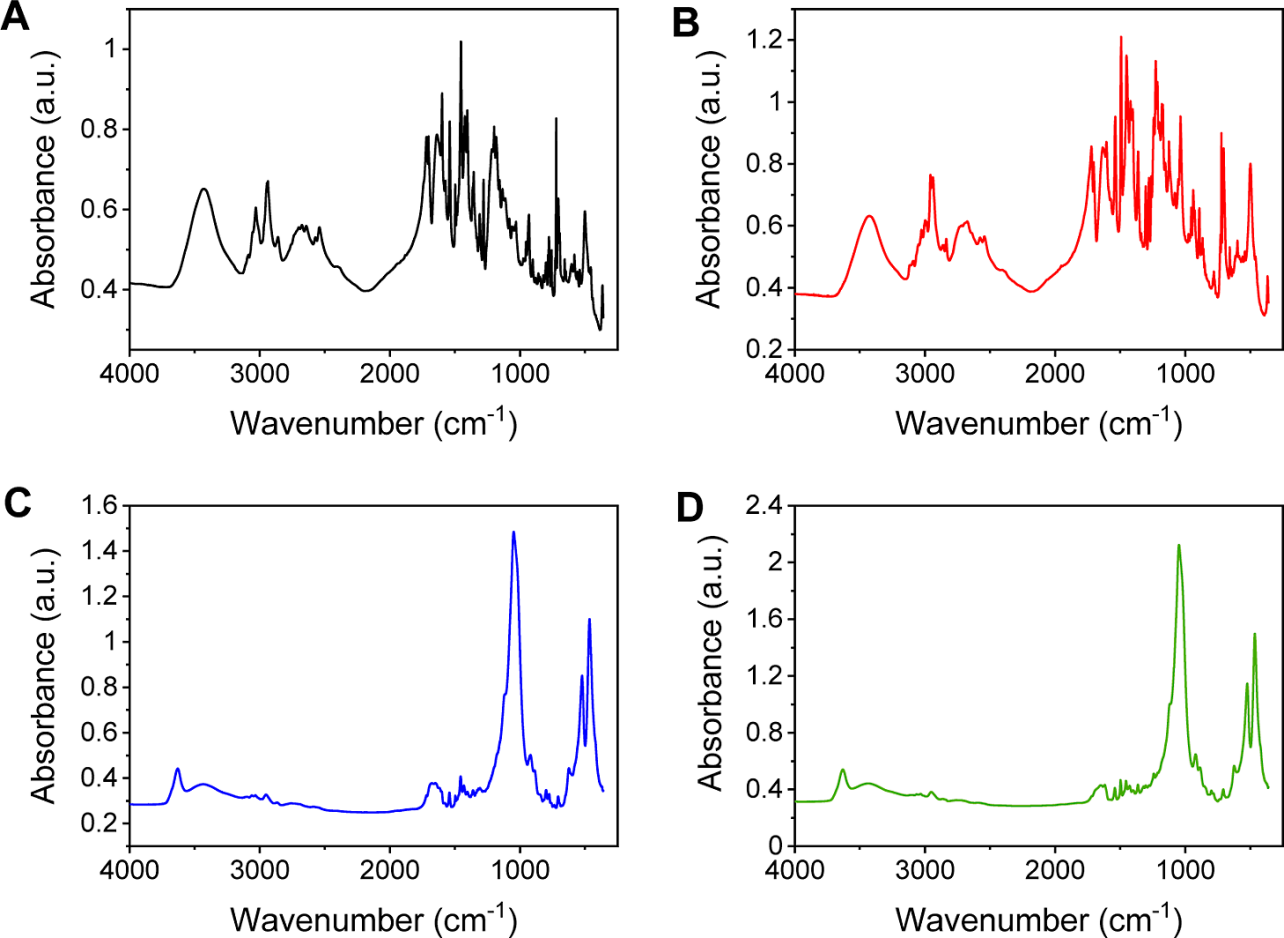
Experimental FT-IR spectra in the 4000–250 cm^–1^ range of (A) 7AI and (B) 5OMe-7AI and the intercalation compounds (C) MMT­(7AI)_5_ and (D) MMT­(5OMe-7AI)_5_.

**Table 3:** Calculated Infrared Frequencies (cm^–1^) of the Optimized 7AI Crystal and Intercalated Structures Compared with Experimental Data

mode^a^	7AI crystal	MMT(7AI)_5_	MMT(7AI)	7AI-crystal _exp_
ν(OH)	3505–3501^b^, 3427–3423^c^	3517–3511[Table-fn t3fn12]	3526–3516[Table-fn t3fn12]	3530–3425
ν(NH)	3273–3271	3318^m^, 3301^n^, 3258–3225^m^	3349	3300–3200
ν(CH)	3261^d^, 3129^e^, 3128^f^, 3103^e^, 3073^g^	3154, 3140–3136^n^, 3121, 3096^d^, 3092–3089^e^	3127^f^, 3092^d^. 3076^e^, 3067^g^	3300–3200, 3114–3085
ν(CH)_arom_	3104^h^, 3092^h^, 3081^i^, 3076^j^, 3071^i^	3105, 3085, 3079–3073	3058_*s*_, 3045–3028	3114–3085,3058–3023
ν(CH_2_)_pipe_	3017^e^, 2990^e^, 2966–2963^g^, 2959^d^, 2956^e^, 2930_s_ ^e^, 2922_s_, 2920_s_ ^e^, 2915_s_ ^d^	3059, 3035, 3020, 3006, 2986–2947	3002–2968^e^, 2952–2949^g^, 2936^d^, 2915^e^, 2909^g^, 2895^d^	2989–2900, 2862
ν(CH_2_)	2979, 2971, 2932_*s*_, 2924,	3013, 3003, 3001–2974, 2953–2948	2993, 2944, 2933	2989–2900
ν(CH_3_)	3016^c^, 2972^c^, 2902_s_ ^c^			2989–2900
ν(C=O)	1813, 1699–1696			1800–1742, 1742–1700
ν(CC)_C_=_C_	1761–1705, 1522–1507		1745–1704	1742–1700, 1570–1540
Ring arom	1598		1722–1647	1570–1540
δ(CH_2_)	1649–1613, 1582, 1530		1639, 1625	1635–1600, 1570–1509
δ(CH_2_)_pipe_	1539, 1483, 1472, 1449, 1436–1378, 1309, 1232		1548–1480^e^, 1475–1467, 1225^g^	1500–1484, 1450–1405
δ(NH)	1505–1503			1500–1484
δ(CH)_arom_	1493, 1302–1287, 1163–1157		1599, 1290, 1238^f^, 1175^e^–1127^g^, 1100^d^	1500–1484, 1280
δ(CH)_alkyl_	1476–1473, 1468		1494–1466	1450
δ(CH)_MeOH_	1480, 1456, 1445, 1116–1113			1450, 1423
δ(OH)	1371–1367^c^, 1331–1323^b^		1626–1604^k^	1352–1309
γ(CH)_indole_	896			
γ(CH)	970–936			931
γ(CH)_arom_	982, 928–911			
γ(OH)	901			902

a Normal vibration modes, as = antisymmetric, s = symmetric, arom = aromatic, indole = indole ring, pipe = piperidine ring, MMT­(7AI) = montmorillonite with only one 7AI molecule per supercell, MMT­(7AI)_5_ with five 7AI molecules per supercell.

b Oxalate moiety.

c Methanol molecule.

d CH group in the para position with respect to the heterocyclic N atom.

e Idem. in the ortho position.

f CH of the small indole ring.

g In the meta position with respect to the heterocyclic N atom.

h CH groups of the aromatic ring in the meta position with respect to the backbone chain.

i Aromatic CH groups in the ortho position with respect to the backbone chain.

j Aromatic CH groups in the para position with respect to the backbone chain.

k Water molecule.

m MOHM of the octahedral sheet of MMT.

n Oriented to the water O atoms.

o Oriented to the mineral surface O atoms.

The ν­(C=O) bands correspond to the stretching mode of the mono-oxalic molecule for the carboxylic (1813 cm^–1^) and the carboxylate (1699–1696 cm^–1^) groups according to previous theoretical and experimental works.^32^ These vibrations are difficult to assign only experimentally due to the overlap with one ν­(C=C) mode. Then, we can assign the oxalic carboxylic ν­(C=O) to the shoulder at the high frequency of the complex multiple band at 1800–1700 cm^–1^. The main band can be assigned to the ν­(C=C) mode at around 1742 cm^–1^. The narrow band at 1700 cm^–1^ can be assigned to the oxalic carboxylate group.

In the intercalated complexes, there are neither oxalate nor methanol molecules; then, the ν­(OH) vibration modes come from the water molecules in the interlayer space and the MOHM’ (M, M’ = Al, Mg) octahedral sheet of MMT. The ν­(NH) of the mono-intercalated model, MMT­(7AI), appears at a higher frequency (3349 cm^–1^) than in the drug crystal (3271 cm^–1^), due to the lack of intramolecular interaction of this N–H group in the interlayer space of MMT. However, in the sample with 5 drug molecules intercalated per supercell, the ν­(NH) frequency decreases due to the increase of interaction of this N–H bond with water molecules (3258–3225 cm^–1^) and the basal O atoms of the mineral surface (3301 cm^–1^). The ν­(CH) frequency of the C–H bond in the para position with respect to the heterocyclic N atom of the six-membered ring in the intercalated drug appears at a lower frequency (3092–3096 cm^–1^) than in the crystal (3261 cm^–1^) due to the changes of the local environment of this group.

## 4 Conclusions

The calculations based on Interface FF accurately reproduce the molecular and crystal structures of both 7-azaindole drugs, validating the use of this FF for the study of these systems. Previously, this FF was also validated for the study of the crystal structure of MMT. Hence, this FF is adequate for the study of the hybrid drug-clay intercalation system.

This atomistic modeling approach has been very useful in interpreting the experimental behavior of the intercalation of these drugs into the MMT interlayer. The intercalation process occurred through a cation-exchange mechanism, and the calculations successfully reproduced the *d*(001) spacing of the intercalation complex, aligning with the cation exchange capacity observed experimentally. Furthermore, this theoretical modeling revealed that this spacing is possible for the incorporation of five 7-azaindole drug molecules per 4 × 2 × 1 montmorillonite supercell, thereby establishing the stoichiometry of the intercalation process.

Besides, in the previous experimental work of this intercalation, a possible monolayer distribution of the drug was tentatively proposed without clear evidence. However, our atomistic calculations have allowed visualization of the possible disposition of 7-azaindole molecules confined in the interlayer of montmorillonite at a complete cation exchange capacity. Our molecular dynamics simulations indicate that they do not form a monolayer but a disordered distribution close to a bilayer structure.

On the other hand, this study successfully calculated the infrared frequencies and assigned them to the main vibration modes of the molecules in their crystal structures. These results were compared with experimental infrared spectroscopic data obtained from solid-state crystals. This analysis enabled the assignment of most of the infrared bands observed experimentally.

The intercalation of these 7-azaindole drugs into the confined interlayer space of montmorillonite is energetically favorable by cation exchange. The loading amount of these drugs is significant, reaching up to 20—21% of the drug per gram of montmorillonite. This amount was higher than previously observed (6.7–7.8%) with halloysite as the host.^8^ This process can be controlled with the pH of the medium. At low pH, these drugs will be stably intercalated into natural montmorillonite, and the delivery can be controlled by increasing the pH of the external medium. This behavior indicates additional stability in the stomach fluids, enhancing the delivery of these drugs in the intestinal zone and increasing their efficiency. This result indicates that these systems can be good candidates for controlled delivery of these drugs.

This work represents a fruitful collaborative investigation that combines experimental and theoretical approaches, predicting a potential application of this hybrid drug–clay complex as a drug delivery system for the treatment of Alzheimer’s disease.

## Supplementary Material



## Data Availability

The data are available throughout the manuscript and supporting files. Additional data related to this work can be available from the corresponding author upon reasonable request to ci.sainz@csic.es

## Notes

Atomic positions of the optimized structures described in this work (PDF).
