# Supplementary material for: Molecular Insights of 7‑Azaindole Drugs and Their Intercalation in the Confined Space of Montmorillonite
Source: ACS Omega. 2025 Dec 3;10(49):60287–97. doi: 10.1021/acsomega.5c06055 (PMC12713467; doi:10.1021/acsomega.5c06055)
Supplement: Supplementary file 1 [file ao5c06055_si_001.pdf]

Molecular insights of 7-azaindole drugs and their intercalation in the confined space of montmorillonite

Ana Borrego-Sánchez[a], Eva M. García-Frutos[b]*, Margarita Darder[b], C. Ignacio Sainz-Díaz[c]*

[a] *Department of Pharmacy and Pharmaceutical Technology and Parasitology, University of Valencia, Avda. Vicente Andrés Estelles SN, 46100, Burjassot, Valencia, Spain.*
[b] *Instituto de Ciencia de Materiales de Madrid (ICMM, CSIC) Campus de Cantoblanco, Madrid, 28049, Spain.*
[c] *Instituto Andaluz de Ciencias de la Tierra (Consejo Superior de Investigaciones Científicas). Av. de las Palmeras 4, 18100-Armilla, Granada, Spain.*
** Corresponding autor: ci.sainz@csic.es, emgfrutos@icmm.csic.es*

SUPPLEMENTARY MATERIAL

Table S1. Atomic coordinates of the unit cell of the optimized crystal structure of the mono-oxalate of 7Al (in fractional coordinates).

cell_length_a	11.7974
cell_length_b	16.2367
cell_length_c	22.8718
cell_angle_alpha	90.0000
cell_angle_beta	90.0000
cell_angle_gamma	90.0000

atom	symbol	x	y	z
C1	C	0.12832	0.29273	0.62851
H2	H	0.07880	0.23648	0.63512
C3	C	0.20474	0.41764	0.63583
C4	C	0.24677	0.49693	0.64535
H5	H	0.21692	0.53491	0.68171
C6	C	0.32730	0.52698	0.60647
H7	H	0.36370	0.58881	0.61215
C8	C	0.36142	0.47843	0.55925
H9	H	0.42296	0.50126	0.52721
N10	N	0.32184	0.40022	0.54967
C11	C	0.24711	0.37615	0.58865
C12	C	0.05223	0.37204	0.71485
H13	H	-0.01970	0.32911	0.71094
H14	H	0.01482	0.43419	0.71781
N15	N	0.10752	0.35372	0.77390
C16	C	0.01352	0.34260	0.82070
H17	H	-0.03954	0.29043	0.80681
H18	H	-0.03880	0.39891	0.81999
C19	C	0.06815	0.32925	0.88045
H20	H	0.11668	0.27114	0.88035
H21	H	0.00028	0.32168	0.91315
C22	C	0.14601	0.40154	0.89646
H23	H	0.18161	0.39328	0.94067
H24	H	0.09603	0.45903	0.89806
C25	C	0.24084	0.40982	0.85088
H26	H	0.29636	0.46275	0.86072

H27	H	0.29516	0.35458	0.85167
C28	C	0.19257	0.42281	0.78962
H29	H	0.14556	0.48118	0.78745
H30	H	0.25876	0.42248	0.75574
C31	C	0.22857	0.23364	0.53639
H32	H	0.23030	0.26518	0.49380
H33	H	0.15627	0.19064	0.53387
C34	C	0.33750	0.18465	0.54500
C35	C	0.39727	0.15561	0.49640
H36	H	0.36707	0.17092	0.45229
C37	C	0.49453	0.10781	0.50237
H38	H	0.53817	0.08411	0.46345
C39	C	0.53574	0.08848	0.55789
H40	H	0.61253	0.05080	0.56268
C41	C	0.47737	0.11696	0.60694
H42	H	0.50714	0.10116	0.65102
C43	C	0.37965	0.16417	0.60023
H44	H	0.33455	0.18474	0.63968
C45	C	0.28112	0.17307	0.75998
C46	C	0.15834	0.13763	0.75706
C47	C	0.37200	0.35844	0.40557
H48	H	0.30816	0.40795	0.40432
H49	H	0.32864	0.29851	0.40198
H50	H	0.42845	0.36569	0.36786
N51	N	0.20279	0.29664	0.58144
H52	H	0.15115	0.29976	0.76953
C53	C	0.12668	0.36380	0.66218
O54	O	0.36170	0.12383	0.75916
O55	O	0.29188	0.24926	0.76234
O56	O	0.13625	0.06559	0.76454
O57	O	0.06883	0.18803	0.74556
H58	H	0.00048	0.15538	0.74476
O59	O	0.44059	0.36325	0.45626
H60	H	0.38878	0.36510	0.48982
C61	C	0.37168	0.70727	0.12851
H62	H	0.42120	0.76352	0.13512
C63	C	0.29526	0.58236	0.13583
C64	C	0.25323	0.50307	0.14535
H65	H	0.28308	0.46509	0.18171
C66	C	0.17270	0.47302	0.10647
H67	H	0.13630	0.41119	0.11215
C68	C	0.13858	0.52157	0.05925
H69	H	0.07704	0.49874	0.02721
N70	N	0.17816	0.59978	0.04967
C71	C	0.25289	0.62385	0.08865
C72	C	0.44777	0.62796	0.21485
H73	H	0.51970	0.67089	0.21094
H74	H	0.48518	0.56581	0.21781
N75	N	0.39248	0.64628	0.27390
C76	C	0.48648	0.65740	0.32070
H77	H	0.53954	0.70957	0.30681
H78	H	0.53880	0.60109	0.31999
C79	C	0.43185	0.67075	0.38045
H80	H	0.38332	0.72886	0.38035
H81	H	0.49972	0.67832	0.41315

C82	C	0.35399	0.59846	0.39646
H83	H	0.31839	0.60672	0.44067
H84	H	0.40397	0.54097	0.39806
C85	C	0.25916	0.59018	0.35088
H86	H	0.20364	0.53725	0.36072
H87	H	0.20484	0.64542	0.35167
C88	C	0.30743	0.57719	0.28962
H89	H	0.35444	0.51882	0.28745
H90	H	0.24124	0.57752	0.25574
C91	C	0.27143	0.76636	0.03639
H92	H	0.26970	0.73482	-0.00620
H93	H	0.34373	0.80936	0.03387
C94	C	0.16250	0.81535	0.04500
C95	C	0.10273	0.84439	-0.00360
H96	H	0.13293	0.82908	-0.04771
C97	C	0.00547	0.89219	0.00237
H98	H	-0.03817	0.91589	-0.03655
C99	C	-0.03574	0.91152	0.05789
H100	H	-0.11253	0.94920	0.06268
C101	C	0.02263	0.88304	0.10694
H102	H	-0.00714	0.89884	0.15102
C103	C	0.12035	0.83583	0.10023
H104	H	0.16545	0.81526	0.13968
C105	C	0.21888	0.82693	0.25998
C106	C	0.34166	0.86237	0.25706
C107	C	0.12800	0.64156	0.90557
H108	H	0.19184	0.59205	0.90432
H109	H	0.17136	0.70149	0.90198
H110	H	0.07155	0.63431	0.86786
N111	N	0.29721	0.70336	0.08144
H112	H	0.34885	0.70024	0.26953
C113	C	0.37332	0.63620	0.16218
O114	O	0.13830	0.87617	0.25916
O115	O	0.20812	0.75074	0.26234
O116	O	0.36375	0.93441	0.26454
O117	O	0.43117	0.81197	0.24556
H118	H	0.49952	0.84462	0.24476
O119	O	0.05941	0.63675	0.95626
H120	H	0.11122	0.63490	0.98982
C121	C	0.87168	0.79273	0.87149
H122	H	0.92120	0.73648	0.86488
C123	C	0.79526	0.91764	0.86417
C124	C	0.75323	0.99693	0.85465
H125	H	0.78308	1.03491	0.81829
C126	C	0.67270	1.02698	0.89353
H127	H	0.63630	1.08881	0.88785
C128	C	0.63858	0.97843	0.94075
H129	H	0.57704	1.00126	0.97279
N130	N	0.67816	0.90022	0.95033
C131	C	0.75289	0.87615	0.91135
C132	C	0.94777	0.87204	0.78515
H133	H	1.01970	0.82911	0.78906
H134	H	0.98518	0.93419	0.78219
N135	N	0.89248	0.85372	0.72610
C136	C	0.98648	0.84260	0.67930

H137	H	1.03954	0.79043	0.69319
H138	H	1.03880	0.89891	0.68001
C139	C	0.93185	0.82925	0.61955
H140	H	0.88332	0.77114	0.61965
H141	H	0.99972	0.82168	0.58685
C142	C	0.85399	0.90154	0.60354
H143	H	0.81839	0.89328	0.55933
H144	H	0.90397	0.95903	0.60194
C145	C	0.75916	0.90982	0.64912
H146	H	0.70364	0.96275	0.63928
H147	H	0.70484	0.85458	0.64833
C148	C	0.80743	0.92281	0.71038
H149	H	0.85444	0.98118	0.71255
H150	H	0.74124	0.92248	0.74426
C151	C	0.77143	0.73364	0.96361
H152	H	0.76970	0.76518	1.00620
H153	H	0.84373	0.69064	0.96613
C154	C	0.66250	0.68465	0.95500
C155	C	0.60273	0.65561	1.00360
H156	H	0.63293	0.67092	1.04771
C157	C	0.50547	0.60781	0.99763
H158	H	0.46183	0.58411	1.03655
C159	C	0.46426	0.58848	0.94211
H160	H	0.38747	0.55080	0.93732
C161	C	0.52263	0.61696	0.89306
H162	H	0.49286	0.60116	0.84898
C163	C	0.62035	0.66417	0.89977
H164	H	0.66545	0.68474	0.86032
C165	C	0.71888	0.67307	0.74002
C166	C	0.84166	0.63763	0.74294
C167	C	0.62800	0.85844	0.09443
H168	H	0.69184	0.90795	0.09568
H169	H	0.67136	0.79851	0.09802
H170	H	0.57155	0.86569	0.13214
N171	N	0.79721	0.79664	0.91856
H172	H	0.84885	0.79976	0.73047
C173	C	0.87332	0.86380	0.83782
O174	O	0.63830	0.62383	0.74084
O175	O	0.70812	0.74926	0.73766
O176	O	0.86375	0.56559	0.73546
O177	O	0.93117	0.68803	0.75444
H178	H	0.99952	0.65538	0.75524
O179	O	0.55941	0.86325	0.04374
H180	H	0.61122	0.86510	0.01018
C181	C	0.62832	0.20727	0.37149
H182	H	0.57880	0.26352	0.36488
C183	C	0.70474	0.08236	0.36417
C184	C	0.74677	0.00307	0.35465
H185	H	0.71692	-0.03491	0.31829
C186	C	0.82730	-0.02698	0.39353
H187	H	0.86370	-0.08881	0.38785
C188	C	0.86142	0.02157	0.44075
H189	H	0.92296	-0.00126	0.47279
N190	N	0.82184	0.09978	0.45033
C191	C	0.74711	0.12385	0.41135

C192	C	0.55223	0.12796	0.28515
H193	H	0.48030	0.17089	0.28906
H194	H	0.51482	0.06581	0.28219
N195	N	0.60752	0.14628	0.22610
C196	C	0.51352	0.15740	0.17930
H197	H	0.46046	0.20957	0.19319
H198	H	0.46120	0.10109	0.18001
C199	C	0.56815	0.17075	0.11955
H200	H	0.61668	0.22886	0.11965
H201	H	0.50028	0.17832	0.08685
C202	C	0.64601	0.09846	0.10354
H203	H	0.68161	0.10672	0.05933
H204	H	0.59603	0.04097	0.10194
C205	C	0.74084	0.09018	0.14912
H206	H	0.79636	0.03725	0.13928
H207	H	0.79516	0.14542	0.14833
C208	C	0.69257	0.07719	0.21038
H209	H	0.64556	0.01882	0.21255
H210	H	0.75876	0.07752	0.24426
C211	C	0.72857	0.26636	0.46361
H212	H	0.73030	0.23482	0.50620
H213	H	0.65627	0.30936	0.46613
C214	C	0.83750	0.31535	0.45500
C215	C	0.89727	0.34439	0.50360
H216	H	0.86707	0.32908	0.54771
C217	C	0.99453	0.39219	0.49763
H218	H	1.03817	0.41589	0.53655
C219	C	1.03574	0.41152	0.44211
H220	H	1.11253	0.44920	0.43732
C221	C	0.97737	0.38304	0.39306
H222	H	1.00714	0.39884	0.34898
C223	C	0.87965	0.33583	0.39977
H224	H	0.83455	0.31526	0.36032
C225	C	0.78112	0.32693	0.24002
C226	C	0.65834	0.36237	0.24294
C227	C	0.87200	0.14156	0.59443
H228	H	0.80816	0.09205	0.59568
H229	H	0.82864	0.20149	0.59802
H230	H	0.92845	0.13431	0.63214
N231	N	0.70279	0.20336	0.41856
H232	H	0.65115	0.20024	0.23047
C233	C	0.62668	0.13620	0.33782
O234	O	0.86170	0.37617	0.24084
O235	O	0.79188	0.25074	0.23766
O236	O	0.63625	0.43441	0.23546
O237	O	0.56883	0.31197	0.25444
H238	H	0.50048	0.34462	0.25524
O239	O	0.94059	0.13675	0.54374
H240	H	0.88878	0.13490	0.51018
C241	C	0.87168	0.70727	0.37149
H242	H	0.92120	0.76352	0.36488
C243	C	0.79526	0.58236	0.36417
C244	C	0.75323	0.50307	0.35465
H245	H	0.78308	0.46509	0.31829
C246	C	0.67270	0.47302	0.39353

H247	H	0.63630	0.41119	0.38785
C248	C	0.63858	0.52157	0.44075
H249	H	0.57704	0.49874	0.47279
N250	N	0.67816	0.59978	0.45033
C251	C	0.75289	0.62385	0.41135
C252	C	0.94777	0.62796	0.28515
H253	H	1.01970	0.67089	0.28906
H254	H	0.98518	0.56581	0.28219
N255	N	0.89248	0.64628	0.22610
C256	C	0.98648	0.65740	0.17930
H257	H	1.03954	0.70957	0.19319
H258	H	1.03880	0.60109	0.18001
C259	C	0.93185	0.67075	0.11955
H260	H	0.88332	0.72886	0.11965
H261	H	0.99972	0.67832	0.08685
C262	C	0.85399	0.59846	0.10354
H263	H	0.81839	0.60672	0.05933
H264	H	0.90397	0.54097	0.10194
C265	C	0.75916	0.59018	0.14912
H266	H	0.70364	0.53725	0.13928
H267	H	0.70484	0.64542	0.14833
C268	C	0.80743	0.57719	0.21038
H269	H	0.85444	0.51882	0.21255
H270	H	0.74124	0.57752	0.24426
C271	C	0.77143	0.76636	0.46361
H272	H	0.76970	0.73482	0.50620
H273	H	0.84373	0.80936	0.46613
C274	C	0.66250	0.81535	0.45500
C275	C	0.60273	0.84439	0.50360
H276	H	0.63293	0.82908	0.54771
C277	C	0.50547	0.89219	0.49763
H278	H	0.46183	0.91589	0.53655
C279	C	0.46426	0.91152	0.44211
H280	H	0.38747	0.94920	0.43732
C281	C	0.52263	0.88304	0.39306
H282	H	0.49286	0.89884	0.34898
C283	C	0.62035	0.83583	0.39977
H284	H	0.66545	0.81526	0.36032
C285	C	0.71888	0.82693	0.24002
C286	C	0.84166	0.86237	0.24294
C287	C	0.62800	0.64156	0.59443
H288	H	0.69184	0.59205	0.59568
H289	H	0.67136	0.70149	0.59802
H290	H	0.57155	0.63431	0.63214
N291	N	0.79721	0.70336	0.41856
H292	H	0.84885	0.70024	0.23047
C293	C	0.87332	0.63620	0.33782
O294	O	0.63830	0.87617	0.24084
O295	O	0.70812	0.75074	0.23766
O296	O	0.86375	0.93441	0.23546
O297	O	0.93117	0.81197	0.25444
H298	H	0.99952	0.84462	0.25524
O299	O	0.55941	0.63675	0.54374
H300	H	0.61122	0.63490	0.51018
C301	C	0.62832	0.29273	0.87149

H302	H	0.57880	0.23648	0.86488
C303	C	0.70474	0.41764	0.86417
C304	C	0.74677	0.49693	0.85465
H305	H	0.71692	0.53491	0.81829
C306	C	0.82730	0.52698	0.89353
H307	H	0.86370	0.58881	0.88785
C308	C	0.86142	0.47843	0.94075
H309	H	0.92296	0.50126	0.97279
N310	N	0.82184	0.40022	0.95033
C311	C	0.74711	0.37615	0.91135
C312	C	0.55223	0.37204	0.78515
H313	H	0.48030	0.32911	0.78906
H314	H	0.51482	0.43419	0.78219
N315	N	0.60752	0.35372	0.72610
C316	C	0.51352	0.34260	0.67930
H317	H	0.46046	0.29043	0.69319
H318	H	0.46120	0.39891	0.68001
C319	C	0.56815	0.32925	0.61955
H320	H	0.61668	0.27114	0.61965
H321	H	0.50028	0.32168	0.58685
C322	C	0.64601	0.40154	0.60354
H323	H	0.68161	0.39328	0.55933
H324	H	0.59603	0.45903	0.60194
C325	C	0.74084	0.40982	0.64912
H326	H	0.79636	0.46275	0.63928
H327	H	0.79516	0.35458	0.64833
C328	C	0.69257	0.42281	0.71038
H329	H	0.64556	0.48118	0.71255
H330	H	0.75876	0.42248	0.74426
C331	C	0.72857	0.23364	0.96361
H332	H	0.73030	0.26518	1.00620
H333	H	0.65627	0.19064	0.96613
C334	C	0.83750	0.18465	0.95500
C335	C	0.89727	0.15561	1.00360
H336	H	0.86707	0.17092	1.04771
C337	C	0.99453	0.10781	0.99763
H338	H	1.03817	0.08411	1.03655
C339	C	1.03574	0.08848	0.94211
H340	H	1.11253	0.05080	0.93732
C341	C	0.97737	0.11696	0.89306
H342	H	1.00714	0.10116	0.84898
C343	C	0.87965	0.16417	0.89977
H344	H	0.83455	0.18474	0.86032
C345	C	0.78112	0.17307	0.74002
C346	C	0.65834	0.13763	0.74294
C347	C	0.87200	0.35844	0.09443
H348	H	0.80816	0.40795	0.09568
H349	H	0.82864	0.29851	0.09802
H350	H	0.92845	0.36569	0.13214
N351	N	0.70279	0.29664	0.91856
H352	H	0.65115	0.29976	0.73047
C353	C	0.62668	0.36380	0.83782
O354	O	0.86170	0.12383	0.74084
O355	O	0.79188	0.24926	0.73766
O356	O	0.63625	0.06559	0.73546

O357	O	0.56883	0.18803	0.75444
H358	H	0.50048	0.15538	0.75524
O359	O	0.94059	0.36325	0.04374
H360	H	0.88878	0.36510	0.01018
C361	C	0.12832	0.20727	0.12851
H362	H	0.07880	0.26352	0.13512
C363	C	0.20474	0.08236	0.13583
C364	C	0.24677	0.00307	0.14535
H365	H	0.21692	-0.03491	0.18171
C366	C	0.32730	-0.02698	0.10647
H367	H	0.36370	-0.08881	0.11215
C368	C	0.36142	0.02157	0.05925
H369	H	0.42296	-0.00126	0.02721
N370	N	0.32184	0.09978	0.04967
C371	C	0.24711	0.12385	0.08865
C372	C	0.05223	0.12796	0.21485
H373	H	-0.01970	0.17089	0.21094
H374	H	0.01482	0.06581	0.21781
N375	N	0.10752	0.14628	0.27390
C376	C	0.01352	0.15740	0.32070
H377	H	-0.03954	0.20957	0.30681
H378	H	-0.03880	0.10109	0.31999
C379	C	0.06815	0.17075	0.38045
H380	H	0.11668	0.22886	0.38035
H381	H	0.00028	0.17832	0.41315
C382	C	0.14601	0.09846	0.39646
H383	H	0.18161	0.10672	0.44067
H384	H	0.09603	0.04097	0.39806
C385	C	0.24084	0.09018	0.35088
H386	H	0.29636	0.03725	0.36072
H387	H	0.29516	0.14542	0.35167
C388	C	0.19257	0.07719	0.28962
H389	H	0.14556	0.01882	0.28745
H390	H	0.25876	0.07752	0.25574
C391	C	0.22857	0.26636	0.03639
H392	H	0.23030	0.23482	-0.00620
H393	H	0.15627	0.30936	0.03387
C394	C	0.33750	0.31535	0.04500
C395	C	0.39727	0.34439	-0.00360
H396	H	0.36707	0.32908	-0.04771
C397	C	0.49453	0.39219	0.00237
H398	H	0.53817	0.41589	-0.03655
C399	C	0.53574	0.41152	0.05789
H400	H	0.61253	0.44920	0.06268
C401	C	0.47737	0.38304	0.10694
H402	H	0.50714	0.39884	0.15102
C403	C	0.37965	0.33583	0.10023
H404	H	0.33455	0.31526	0.13968
C405	C	0.28112	0.32693	0.25998
C406	C	0.15834	0.36237	0.25706
C407	C	0.37200	0.14156	0.90557
H408	H	0.30816	0.09205	0.90432
H409	H	0.32864	0.20149	0.90198
H410	H	0.42845	0.13431	0.86786
N411	N	0.20279	0.20336	0.08144

H412	H	0.15115	0.20024	0.26953
C413	C	0.12668	0.13620	0.16218
O414	O	0.36170	0.37617	0.25916
O415	O	0.29188	0.25074	0.26234
O416	O	0.13625	0.43441	0.26454
O417	O	0.06883	0.31197	0.24556
H418	H	0.00048	0.34462	0.24476
O419	O	0.44059	0.13675	0.95626
H420	H	0.38878	0.13490	0.98982
C421	C	0.37168	0.79273	0.62851
H422	H	0.42120	0.73648	0.63512
C423	C	0.29526	0.91764	0.63583
C424	C	0.25323	0.99693	0.64535
H425	H	0.28308	1.03491	0.68171
C426	C	0.17270	1.02698	0.60647
H427	H	0.13630	1.08881	0.61215
C428	C	0.13858	0.97843	0.55925
H429	H	0.07704	1.00126	0.52721
N430	N	0.17816	0.90022	0.54967
C431	C	0.25289	0.87615	0.58865
C432	C	0.44777	0.87204	0.71485
H433	H	0.51970	0.82911	0.71094
H434	H	0.48518	0.93419	0.71781
N435	N	0.39248	0.85372	0.77390
C436	C	0.48648	0.84260	0.82070
H437	H	0.53954	0.79043	0.80681
H438	H	0.53880	0.89891	0.81999
C439	C	0.43185	0.82925	0.88045
H440	H	0.38332	0.77114	0.88035
H441	H	0.49972	0.82168	0.91315
C442	C	0.35399	0.90154	0.89646
H443	H	0.31839	0.89328	0.94067
H444	H	0.40397	0.95903	0.89806
C445	C	0.25916	0.90982	0.85088
H446	H	0.20364	0.96275	0.86072
H447	H	0.20484	0.85458	0.85167
C448	C	0.30743	0.92281	0.78962
H449	H	0.35444	0.98118	0.78745
H450	H	0.24124	0.92248	0.75574
C451	C	0.27143	0.73364	0.53639
H452	H	0.26970	0.76518	0.49380
H453	H	0.34373	0.69064	0.53387
C454	C	0.16250	0.68465	0.54500
C455	C	0.10273	0.65561	0.49640
H456	H	0.13293	0.67092	0.45229
C457	C	0.00547	0.60781	0.50237
H458	H	-0.03817	0.58411	0.46345
C459	C	-0.03574	0.58848	0.55789
H460	H	-0.11253	0.55080	0.56268
C461	C	0.02263	0.61696	0.60694
H462	H	-0.00714	0.60116	0.65102
C463	C	0.12035	0.66417	0.60023
H464	H	0.16545	0.68474	0.63968
C465	C	0.21888	0.67307	0.75998
C466	C	0.34166	0.63763	0.75706

C467	C	0.12800	0.85844	0.40557
H468	H	0.19184	0.90795	0.40432
H469	H	0.17136	0.79851	0.40198
H470	H	0.07155	0.86569	0.36786
N471	N	0.29721	0.79664	0.58144
H472	H	0.34885	0.79976	0.76953
C473	C	0.37332	0.86380	0.66218
O474	O	0.13830	0.62383	0.75916
O475	O	0.20812	0.74926	0.76234
O476	O	0.36375	0.56559	0.76454
O477	O	0.43117	0.68803	0.74556
H478	H	0.49952	0.65538	0.74476
O479	O	0.05941	0.86325	0.45626
H480	H	0.11122	0.86510	0.48982

Table S2. Atomic coordinates of the asymmetric unit of the optimized crystal structure of the mono-oxalate of 5OMe-7AI (in fractional coordinates).

cell_length_a	8.2251
cell_length_b	23.9306
cell_length_c	11.6828
cell_angle_alpha	90.0000
cell_angle_beta	110.5315
cell_angle_gamma	90.0000

atom	symbol	x	y	z
C1	C	0.63778	0.41534	0.62252
C2	C	0.82330	0.35653	0.60380
H2	H	0.95062	0.33806	0.61805
C3	C	0.66258	0.33939	0.52336
C4	C	0.54193	0.37819	0.53683
C5	C	0.36435	0.38822	0.48257
H5	H	0.28451	0.35978	0.41144
C6	C	0.29445	0.43457	0.52120
C7	C	0.41119	0.46906	0.61188
H7	H	0.36581	0.50652	0.64610
C8	C	0.00315	0.41137	0.39166
H8A	H	0.03047	0.40980	0.30605
H8B	H	-0.13046	0.42632	0.37104
H8C	H	0.01350	0.36904	0.43028
C9	C	0.64412	0.29042	0.43943
H9A	H	0.77038	0.28003	0.43244
H9B	H	0.55958	0.30216	0.34616
C10	C	0.67815	0.21395	0.59500
H10A	H	0.67281	0.24611	0.66086
H10B	H	0.81246	0.20973	0.59755
C11	C	0.60866	0.15751	0.61829
H11A	H	0.69538	0.14221	0.70794
H11B	H	0.47940	0.16275	0.62547
C12	C	0.59781	0.11527	0.51695
H12A	H	0.72942	0.10662	0.51641
H12B	H	0.54623	0.07499	0.53530
C13	C	0.48399	0.13814	0.39167
H13A	H	0.34949	0.14399	0.38856
H13B	H	0.47783	0.10844	0.31810
C14	C	0.55517	0.19339	0.36451

H14A	H	0.68668	0.18834	0.36114
H14B	H	0.47237	0.21233	0.27837
C15	C	0.96235	0.44229	0.73680
H15A	H	0.91178	0.48525	0.71802
H15B	H	1.06210	0.43887	0.69394
C16	C	1.05175	0.43618	0.87478
C17	C	1.16001	0.47979	0.93752
H17	H	1.17426	0.51668	0.88584
C18	C	1.24694	0.47828	1.06336
H18	H	1.32855	0.51360	1.11046
C19	C	1.22760	0.43216	1.13045
H19	H	1.29475	0.43107	1.22999
C20	C	1.12142	0.38801	1.06951
H20	H	1.10636	0.35141	1.12170
C21	C	1.03570	0.39010	0.94324
H21	H	0.95471	0.35457	0.89713
N1	N	0.81697	0.40438	0.66948
N2	N	0.58526	0.46016	0.66405
N3	N	0.56849	0.23606	0.46728
H3N	H	0.44703	0.24458	0.46742
O1	O	0.11977	0.44856	0.47646
C22	C	0.10887	0.25415	0.37162
C23	C	0.17345	0.25582	0.51377
O2	O	-0.04187	0.24721	0.30990
O3	O	0.22087	0.26109	0.30972
H3	H	0.15676	0.25604	0.22357
O4	O	0.06480	0.25106	0.56483
O5	O	0.33157	0.26184	0.56897

Table S3. Cell parameters and atomic coordinates of the 4x2x1 supercell of MMT (in fractional coordinates).

cell_length_a	20.6544
cell_length_b	17.8998
cell_length_c	12.3063
cell_angle_alpha	92.8781
cell_angle_beta	112.2055
cell_angle_gamma	90.1028

atom	symbol	x	y	z
Mg1	Mg	0.12830	0.07444	0.99696
Al2	Al	0.25381	0.32431	0.99765
Si3	Si	0.21894	0.49510	1.23156
Si4	Si	0.09359	0.24480	1.23005
Si5	Si	0.22209	0.15958	1.23186
Si6	Si	0.09693	0.40932	1.23091
O7	O	0.18015	-0.00605	1.08890
O8	O	0.05532	0.24362	1.08776
O9	O	0.19613	0.14962	1.08920
O10	O	0.07100	0.39985	1.08810
O11	O	0.07132	0.08749	1.09085
O12	O	0.19644	0.33754	1.09106
O13	O	0.05738	0.18251	1.28758
O14	O	0.18226	0.43359	1.28908

O15	O	0.21167	0.07976	1.28889
O16	O	0.08675	0.32934	1.28741
O17	O	0.17829	0.22669	1.27012
O18	O	0.05374	0.47658	1.27000
Al19	Al	0.12888	0.40747	0.99623
Al20	Al	0.00354	0.15746	0.99539
Si21	Si	0.03839	-0.01308	0.76117
Si22	Si	0.16381	0.23638	0.76278
Si23	Si	0.03535	0.32180	0.76123
Si24	Si	0.16083	0.07191	0.76292
O25	O	0.07720	0.48816	0.90368
O26	O	0.20235	0.23821	0.90529
O27	O	0.06099	0.33235	0.90377
O28	O	0.18634	0.08205	0.90558
O29	O	0.18605	0.39411	0.90250
O30	O	0.06105	0.14408	0.90243
O31	O	0.20037	0.29781	0.70508
O32	O	0.07548	0.04757	0.70242
O33	O	0.04457	0.40185	0.70352
O34	O	0.17143	0.15180	0.70625
O35	O	0.07912	0.25487	0.72261
O36	O	0.20462	0.00497	0.72406
H37	H	0.05343	0.04553	1.11062
H38	H	0.18273	0.29725	1.12149
H39	H	0.20043	0.43411	0.87238
H40	H	0.07474	0.18362	0.87102
Na41	Na	0.12672	0.08168	0.36950
Al42	Al	0.37888	0.07449	0.99763
Al43	Al	0.50387	0.32466	0.99773
Si44	Si	0.46927	0.49552	1.23317
Si45	Si	0.34400	0.24512	1.23272
Si46	Si	0.47245	0.15991	1.23224
Si47	Si	0.34741	0.40990	1.23246
O48	O	0.43090	-0.00620	1.08975
O49	O	0.30572	0.24357	1.08984
O50	O	0.44639	0.15012	1.08959
O51	O	0.32135	0.39974	1.08973
O52	O	0.32182	0.08746	1.09167
O53	O	0.44706	0.33803	1.09213
O54	O	0.30757	0.18359	1.29076
O55	O	0.43270	0.43402	1.29091
O56	O	0.46456	0.07994	1.29083
O57	O	0.33762	0.32998	1.28944
O58	O	0.42863	0.22656	1.27164
O59	O	0.30376	0.47733	1.27132
Al60	Al	0.37908	0.40778	0.99756
Al61	Al	0.25404	0.15745	0.99739
Si62	Si	0.28906	-0.01322	0.76247
Si63	Si	0.41371	0.23702	0.76259
Si64	Si	0.28567	0.32230	0.76295
Si65	Si	0.41115	0.07251	0.76276
O66	O	0.32705	0.48843	0.90528
O67	O	0.45213	0.23845	0.90537
O68	O	0.31172	0.33193	0.90574
O69	O	0.43649	0.08256	0.90533

O70	O	0.43619	0.39480	0.90370
O71	O	0.31092	0.14430	0.90295
O72	O	0.45006	0.29843	0.70458
O73	O	0.32551	0.04881	0.70468
O74	O	0.29511	0.40182	0.70550
O75	O	0.41997	0.15242	0.70463
O76	O	0.32906	0.25468	0.72453
O77	O	0.45409	0.00542	0.72267
H78	H	0.30662	0.04735	1.12028
H79	H	0.43439	0.29900	1.12609
H80	H	0.44944	0.43568	0.87404
H81	H	0.32435	0.18432	0.87187
Mg82	Mg	0.62850	0.07460	0.99743
Al83	Al	0.75355	0.32403	0.99619
Si84	Si	0.71989	0.49522	1.23260
Si85	Si	0.59461	0.24514	1.23279
Si86	Si	0.72279	0.15956	1.23257
Si87	Si	0.59751	0.40999	1.23260
O88	O	0.67999	-0.00587	1.08978
O89	O	0.55559	0.24401	1.09005
O90	O	0.69632	0.14979	1.08943
O91	O	0.57186	0.39985	1.08981
O92	O	0.57181	0.08792	1.09146
O93	O	0.69706	0.33745	1.09130
O94	O	0.55754	0.18467	1.29170
O95	O	0.68312	0.43422	1.29132
O96	O	0.71430	0.07997	1.29187
O97	O	0.58867	0.33002	1.29003
O98	O	0.67924	0.22668	1.27189
O99	O	0.55400	0.47717	1.27149
Al100	Al	0.62931	0.40756	0.99716
Al101	Al	0.50416	0.15784	0.99749
Si102	Si	0.53864	-0.01338	0.76158
Si103	Si	0.66304	0.23600	0.76166
Si104	Si	0.53543	0.32250	0.76221
Si105	Si	0.66075	0.07154	0.76267
O106	O	0.57741	0.48826	0.90507
O107	O	0.70171	0.23794	0.90417
O108	O	0.56151	0.33231	0.90517
O109	O	0.68565	0.08225	0.90494
O110	O	0.68591	0.39440	0.90255
O111	O	0.56099	0.14453	0.90346
O112	O	0.69979	0.29677	0.70285
O113	O	0.57559	0.04668	0.70163
O114	O	0.54516	0.40235	0.70531
O115	O	0.66718	0.15124	0.70236
O116	O	0.57857	0.25520	0.72261
O117	O	0.70400	0.00527	0.72150
H118	H	0.55852	0.04756	1.12193
H119	H	0.68286	0.29649	1.11965
H120	H	0.69994	0.43431	0.87180
H121	H	0.57564	0.18448	0.87399
Na122	Na	0.56673	0.10329	0.51129
Mg123	Mg	0.87804	0.07452	0.99526
Al124	Al	1.00333	0.32425	0.99607

Si125	Si	0.96936	0.49524	1.23123
Si126	Si	0.84426	0.24459	1.22997
Si127	Si	0.97156	0.15937	1.22945
Si128	Si	0.84770	0.40940	1.23082
O129	O	0.93025	-0.00586	1.08734
O130	O	0.80528	0.24354	1.08815
O131	O	0.94559	0.14992	1.08714
O132	O	0.82128	0.39947	1.08830
O133	O	0.82177	0.08755	1.08994
O134	O	0.94647	0.33743	1.09040
O135	O	0.80789	0.18577	1.29188
O136	O	0.93314	0.43358	1.28940
O137	O	0.96276	0.07985	1.28774
O138	O	0.83914	0.32928	1.28873
O139	O	0.92874	0.22650	1.26971
O140	O	0.80433	0.47611	1.27138
Al141	Al	0.87879	0.40739	0.99585
Al142	Al	0.75371	0.15734	0.99633
Si143	Si	0.78845	-0.01325	0.76012
Si144	Si	0.91325	0.23619	0.76112
Si145	Si	0.78492	0.32195	0.76120
Si146	Si	0.91001	0.07215	0.76011
O147	O	0.82702	0.48828	0.90401
O148	O	0.95204	0.23825	0.90335
O149	O	0.81113	0.33202	0.90379
O150	O	0.93541	0.08259	0.90300
O151	O	0.93565	0.39434	0.90156
O152	O	0.81013	0.14407	0.90148
O153	O	0.94981	0.29687	0.70131
O154	O	0.82471	0.04721	0.70012
O155	O	0.79326	0.40186	0.70287
O156	O	0.91851	0.15213	0.70190
O157	O	0.82883	0.25586	0.72051
O158	O	0.95383	0.00532	0.72106
H159	H	0.80653	0.04683	1.11696
H160	H	0.93208	0.29646	1.11837
H161	H	0.94949	0.43567	0.87377
H162	H	0.82392	0.18377	0.87052
Na163	Na	0.87661	0.29196	0.49289
Al164	Al	0.12844	0.57442	0.99643
Al165	Al	0.25378	0.82459	0.99687
Si166	Si	0.21909	0.99526	1.23154
Si167	Si	0.09388	0.74545	1.23156
Si168	Si	0.22195	0.65983	1.23115
Si169	Si	0.09700	0.90991	1.23049
O170	O	0.18049	0.49368	1.08864
O171	O	0.05549	0.74368	1.08867
O172	O	0.19600	0.65005	1.08848
O173	O	0.07153	0.89948	1.08843
O174	O	0.07140	0.58731	1.09037
O175	O	0.19685	0.83792	1.09100
O176	O	0.05761	0.68397	1.28966
O177	O	0.18216	0.93455	1.28969
O178	O	0.21265	0.57991	1.28856
O179	O	0.08745	0.83032	1.28822

O180	O	0.17857	0.72716	1.26995
O181	O	0.05386	0.97802	1.26894
Al182	Al	0.12895	0.90761	0.99611
Al183	Al	0.00373	0.65748	0.99643
Si184	Si	0.03847	0.48681	0.76095
Si185	Si	0.16368	0.73692	0.76128
Si186	Si	0.03539	0.82232	0.76163
Si187	Si	0.16078	0.57236	0.76152
O188	O	0.07726	0.98836	0.90423
O189	O	0.20206	0.73839	0.90422
O190	O	0.06161	0.83164	0.90444
O191	O	0.18604	0.58247	0.90418
O192	O	0.18626	0.89468	0.90258
O193	O	0.06047	0.64422	0.90178
O194	O	0.20045	0.79825	0.70366
O195	O	0.07515	0.54805	0.70249
O196	O	0.04455	0.90206	0.70414
O197	O	0.16984	0.65220	0.70375
O198	O	0.07900	0.75502	0.72275
O199	O	0.20393	0.50487	0.72274
H200	H	0.05640	0.54738	1.11960
H201	H	0.18388	0.79879	1.12438
H202	H	0.19947	0.93558	0.87293
H203	H	0.07380	0.68417	0.87039
Mg204	Mg	0.37850	0.57453	0.99767
Al205	Al	0.50408	0.82421	0.99768
Si206	Si	0.46972	0.99509	1.23250
Si207	Si	0.34423	0.74516	1.23160
Si208	Si	0.47237	0.65955	1.23286
Si209	Si	0.34736	0.90982	1.23222
O210	O	0.43021	0.49413	1.09004
O211	O	0.30536	0.74384	1.08902
O212	O	0.44622	0.64965	1.08972
O213	O	0.32170	0.89982	1.08932
O214	O	0.32145	0.58784	1.09110
O215	O	0.44710	0.83760	1.09177
O216	O	0.30777	0.68348	1.29015
O217	O	0.43275	0.93427	1.29060
O218	O	0.46261	0.58001	1.29076
O219	O	0.33771	0.82971	1.28893
O220	O	0.42877	0.72694	1.27130
O221	O	0.30376	0.97718	1.27067
Al222	Al	0.37945	0.90754	0.99724
Al223	Al	0.25383	0.65772	0.99645
Si224	Si	0.28864	0.48662	0.76192
Si225	Si	0.41362	0.73621	0.76268
Si226	Si	0.28572	0.82244	0.76204
Si227	Si	0.41033	0.57185	0.76275
O228	O	0.32770	0.98821	0.90508
O229	O	0.45234	0.73807	0.90533
O230	O	0.31172	0.83219	0.90490
O231	O	0.43617	0.58207	0.90567
O232	O	0.43643	0.89427	0.90320
O233	O	0.31117	0.64430	0.90328
O234	O	0.45033	0.79708	0.70432

O235	O	0.32527	0.54734	0.70313
O236	O	0.29569	0.90227	0.70539
O237	O	0.41949	0.65156	0.70475
O238	O	0.32889	0.75492	0.72268
O239	O	0.45414	0.50526	0.72323
H240	H	0.30765	0.54720	1.12028
H241	H	0.43371	0.79745	1.12290
H242	H	0.45072	0.93426	0.87295
H243	H	0.32539	0.68409	0.87296
Na244	Na	0.37499	0.62629	0.46304
Al245	Al	0.62881	0.57444	0.99755
Al246	Al	0.75372	0.82481	0.99636
Si247	Si	0.71979	0.99547	1.23261
Si248	Si	0.59428	0.74498	1.23278
Si249	Si	0.72261	0.65991	1.23195
Si250	Si	0.59800	0.90972	1.23246
O251	O	0.68093	0.49374	1.08976
O252	O	0.55590	0.74347	1.08985
O253	O	0.69652	0.65004	1.08930
O254	O	0.57174	0.89949	1.08965
O255	O	0.57190	0.58742	1.09174
O256	O	0.69721	0.83801	1.09139
O257	O	0.55770	0.68381	1.29086
O258	O	0.68342	0.93400	1.29092
O259	O	0.71475	0.58008	1.29053
O260	O	0.58828	0.82989	1.28958
O261	O	0.67895	0.72656	1.27130
O262	O	0.55423	0.97667	1.27174
Al263	Al	0.62916	0.90779	0.99698
Al264	Al	0.50407	0.65739	0.99763
Si265	Si	0.53866	0.48690	0.76225
Si266	Si	0.66368	0.73683	0.76164
Si267	Si	0.53551	0.82218	0.76242
Si268	Si	0.66077	0.57238	0.76241
O269	O	0.57687	0.98845	0.90489
O270	O	0.70187	0.73838	0.90445
O271	O	0.56182	0.83169	0.90536
O272	O	0.68627	0.58240	0.90499
O273	O	0.68590	0.89488	0.90254
O274	O	0.56084	0.64424	0.90304
O275	O	0.70006	0.79780	0.70320
O276	O	0.57522	0.54855	0.70422
O277	O	0.54428	0.90175	0.70459
O278	O	0.66921	0.65210	0.70420
O279	O	0.57889	0.75490	0.72262
O280	O	0.70363	0.50517	0.72263
H281	H	0.55694	0.54761	1.12131
H282	H	0.68409	0.79864	1.12404
H283	H	0.70060	0.93650	0.87626
H284	H	0.57419	0.68422	0.87181
Mg285	Mg	0.87836	0.57441	0.99652
Al286	Al	1.00360	0.82453	0.99646
Si287	Si	0.96875	0.99543	1.22972
Si288	Si	0.84455	0.74556	1.23113
Si289	Si	0.97240	0.65950	1.23172

Si290	Si	0.84762	0.91027	1.23046
O291	O	0.93021	0.49385	1.08834
O292	O	0.80545	0.74399	1.08873
O293	O	0.94610	0.64946	1.08869
O294	O	0.82154	0.89987	1.08810
O295	O	0.82176	0.58770	1.09067
O296	O	0.94674	0.83807	1.09058
O297	O	0.80791	0.68529	1.29152
O298	O	0.93287	0.93415	1.28845
O299	O	0.96389	0.57966	1.29048
O300	O	0.83840	0.83034	1.28834
O301	O	0.92896	0.72656	1.27120
O302	O	0.80427	0.97675	1.27102
Al303	Al	0.87893	0.90780	0.99542
Al304	Al	0.75398	0.65777	0.99661
Si305	Si	0.78803	0.48675	0.76096
Si306	Si	0.91320	0.73679	0.76252
Si307	Si	0.78518	0.82257	0.76135
Si308	Si	0.90981	0.57203	0.76113
O309	O	0.82719	0.98831	0.90293
O310	O	0.95206	0.73820	0.90460
O311	O	0.81135	0.83222	0.90391
O312	O	0.93576	0.58218	0.90414
O313	O	0.93597	0.89429	0.90147
O314	O	0.81088	0.64421	0.90267
O315	O	0.95016	0.79677	0.70316
O316	O	0.82482	0.54680	0.70127
O317	O	0.79426	0.90184	0.70274
O318	O	0.91790	0.65225	0.70278
O319	O	0.82879	0.75555	0.72232
O320	O	0.95392	0.50538	0.72180
H321	H	0.80809	0.54726	1.12045
H322	H	0.93303	0.79809	1.12142
H323	H	0.95034	0.93578	0.87446
H324	H	0.82477	0.68405	0.87191
Na325	Na	0.86301	0.67982	0.49980
O326	O	0.05775	0.07597	0.49024
H327	H	0.05622	0.06423	0.56661
H328	H	0.00749	0.07524	0.44160
O329	O	0.27887	0.66306	0.51943
H330	H	0.28316	0.69582	0.58848
H331	H	0.23286	0.63990	0.50251
O332	O	0.49802	0.64768	0.54965
H333	H	0.52780	0.69255	0.58223
H334	H	0.53162	0.60870	0.58594
O335	O	0.45462	0.14147	0.51234
H336	H	0.44455	0.14916	0.58408
H337	H	0.40812	0.14913	0.45240
O338	O	0.22425	0.12077	0.54286
H339	H	0.20868	0.14348	0.60306
H340	H	0.26828	0.09887	0.59317
O341	O	0.67427	0.08314	0.47984
H342	H	0.72060	0.07583	0.54079
H343	H	0.68576	0.07922	0.40902
O344	O	0.81862	0.80458	0.48747

H345	H	0.81038	0.84340	0.53972
H346	H	0.81518	0.83435	0.42057
O347	O	0.78175	0.37051	0.48356
H348	H	0.77554	0.39184	0.55423
H349	H	0.74065	0.39039	0.42158
O350	O	0.96027	0.60627	0.50100
H351	H	0.96744	0.59149	0.42819
H352	H	1.00526	0.59264	0.56050
O353	O	1.97812	0.35086	0.48018
H354	H	1.97994	0.37385	0.41044
H355	H	2.02142	0.37159	0.54108

Table S4. Cell parameters and atomic coordinates of the 4x2x1 supercell of MMT with one 7AI molecule intercalated (in fractional coordinates).

cell_length_a	20.6497
cell_length_b	17.9007
cell_length_c	14.9801
cell_angle_alpha	104.6061
cell_angle_beta	83.7013
cell_angle_gamma	90.0989

atom	symbol	x	y	z
Mg1	Mg	0.12862	0.09257	0.51317
Al2	Al	0.25400	0.34246	0.51365
Si3	Si	0.15170	0.54434	0.69986
Si4	Si	0.02716	0.29344	0.69827
Si5	Si	0.15511	0.20834	0.69791
Si6	Si	0.02992	0.45833	0.69958
O7	O	0.15402	0.02445	0.58629
O8	O	0.02915	0.27391	0.58616
O9	O	0.17007	0.17984	0.58561
O10	O	0.04511	0.42966	0.58671
O11	O	0.04501	0.11848	0.58816
O12	O	0.16983	0.36767	0.58791
O13	O	-0.02542	0.23915	0.74460
O14	O	0.09848	0.49022	0.74534
O15	O	0.12852	0.13697	0.74454
O16	O	0.00463	0.38576	0.74559
O17	O	0.10041	0.28014	0.72890
O18	O	-0.02485	0.53046	0.73102
Al19	Al	0.12929	0.42556	0.51365
Al20	Al	0.00410	0.17567	0.51339
Si21	Si	0.10629	-0.02626	0.32744
Si22	Si	0.23156	0.22363	0.32758
Si23	Si	0.10325	0.30931	0.32783
Si24	Si	0.22816	0.05903	0.32734
O25	O	0.10409	0.49418	0.44088
O26	O	0.22920	0.24424	0.44037
O27	O	0.08809	0.33815	0.44069
O28	O	0.21280	0.08805	0.44046
O29	O	0.21339	0.40024	0.43931
O30	O	0.08796	0.14989	0.43907
O31	O	0.28487	0.27782	0.28220
O32	O	0.15975	0.02685	0.28074

O33	O	0.12901	0.38180	0.28265
O34	O	0.25454	0.13150	0.28245
O35	O	0.15817	0.23725	0.29663
O36	O	0.28316	-0.01328	0.29746
H37	H	0.02186	0.08024	0.60708
H38	H	0.14740	0.33078	0.61075
H39	H	0.23623	0.43648	0.41577
H40	H	0.11072	0.18495	0.41358
Al41	Al	0.37892	0.09263	0.51417
Al42	Al	0.50382	0.34292	0.51455
Si43	Si	0.40159	0.54482	0.70029
Si44	Si	0.27672	0.29419	0.69926
Si45	Si	0.40498	0.20886	0.69985
Si46	Si	0.28001	0.45896	0.69938
O47	O	0.40431	0.02415	0.58719
O48	O	0.27933	0.27374	0.58638
O49	O	0.41993	0.18044	0.58698
O50	O	0.29496	0.43002	0.58659
O51	O	0.29457	0.11805	0.58794
O52	O	0.41976	0.36859	0.58872
O53	O	0.22288	0.24119	0.74604
O54	O	0.34862	0.49032	0.74548
O55	O	0.37871	0.13665	0.74486
O56	O	0.25393	0.38635	0.74427
O57	O	0.35000	0.28101	0.73044
O58	O	0.22507	0.53107	0.73055
Al59	Al	0.37925	0.42607	0.51407
Al60	Al	0.25432	0.17544	0.51290
Si61	Si	0.35667	-0.02607	0.32811
Si62	Si	0.48158	0.22404	0.32852
Si63	Si	0.35353	0.30981	0.32812
Si64	Si	0.47869	0.05966	0.32880
O65	O	0.35403	0.49475	0.44113
O66	O	0.47888	0.24452	0.44136
O67	O	0.33838	0.33836	0.44101
O68	O	0.46325	0.08839	0.44153
O69	O	0.46346	0.40068	0.43997
O70	O	0.33853	0.14981	0.43908
O71	O	0.53528	0.27738	0.28275
O72	O	0.41014	0.02779	0.28269
O73	O	0.37955	0.38223	0.28295
O74	O	0.50491	0.13201	0.28364
O75	O	0.40828	0.23740	0.29760
O76	O	0.53341	-0.01262	0.29773
H77	H	0.27115	0.08146	0.61004
H78	H	0.39729	0.33404	0.61554
H79	H	0.48540	0.43813	0.41769
H80	H	0.36078	0.18428	0.41187
Mg81	Mg	0.62868	0.09295	0.51452
Al82	Al	0.75404	0.34257	0.51478
Si83	Si	0.65183	0.54453	0.70052
Si84	Si	0.52678	0.29453	0.70011
Si85	Si	0.65473	0.20922	0.69952
Si86	Si	0.52971	0.45915	0.70015
O87	O	0.65371	0.02468	0.58737

O88	O	0.52903	0.27443	0.58731
O89	O	0.66999	0.18010	0.58716
O90	O	0.54520	0.43024	0.58730
O91	O	0.54507	0.11873	0.58869
O92	O	0.66999	0.36810	0.58912
O93	O	0.47344	0.24096	0.74610
O94	O	0.59850	0.49072	0.74596
O95	O	0.62947	0.13721	0.74616
O96	O	0.50377	0.38668	0.74510
O97	O	0.60040	0.28169	0.73068
O98	O	0.47525	0.53153	0.73043
Al99	Al	0.62946	0.42577	0.51465
Al100	Al	0.50425	0.17605	0.51428
Si101	Si	0.60656	-0.02585	0.32880
Si102	Si	0.73156	0.22359	0.32986
Si103	Si	0.60342	0.30958	0.32916
Si104	Si	0.72858	0.05927	0.32979
O105	O	0.60421	0.49413	0.44155
O106	O	0.72895	0.24431	0.44202
O107	O	0.58827	0.33826	0.44180
O108	O	0.71276	0.08843	0.44215
O109	O	0.71340	0.39986	0.43991
O110	O	0.58848	0.15059	0.44022
O111	O	0.78453	0.27791	0.28392
O112	O	0.66024	0.02752	0.28210
O113	O	0.62948	0.38167	0.28381
O114	O	0.75548	0.13152	0.28467
O115	O	0.65818	0.23665	0.29754
O116	O	0.78332	-0.01285	0.29794
H117	H	0.52276	0.08300	0.61369
H118	H	0.64751	0.33195	0.61337
H119	H	0.73631	0.43641	0.41686
H120	H	0.61147	0.18750	0.41854
Mg121	Mg	0.87850	0.09273	0.51436
Al122	Al	1.00377	0.34241	0.51360
Si123	Si	0.90195	0.54463	0.70016
Si124	Si	0.77734	0.29431	0.69946
Si125	Si	0.90561	0.20838	0.69883
Si126	Si	0.78018	0.45897	0.69990
O127	O	0.90383	0.02437	0.58700
O128	O	0.77959	0.27407	0.58724
O129	O	0.91981	0.18015	0.58648
O130	O	0.79506	0.43018	0.58728
O131	O	0.79478	0.11817	0.58957
O132	O	0.91997	0.36827	0.58872
O133	O	0.72406	0.24010	0.74515
O134	O	0.84853	0.49115	0.74627
O135	O	0.87902	0.13644	0.74440
O136	O	0.75475	0.38648	0.74524
O137	O	0.85063	0.28051	0.73077
O138	O	0.72513	0.53102	0.73177
Al139	Al	0.87929	0.42598	0.51435
Al140	Al	0.75427	0.17575	0.51466
Si141	Si	0.85638	-0.02606	0.32920
Si142	Si	0.98131	0.22350	0.32812

Si143	Si	0.85336	0.30995	0.32963
Si144	Si	0.97810	0.05917	0.32887
O145	O	0.85376	0.49446	0.44148
O146	O	0.97865	0.24409	0.44064
O147	O	0.83826	0.33833	0.44194
O148	O	0.96233	0.08856	0.44127
O149	O	0.96310	0.40036	0.43958
O150	O	0.83800	0.14989	0.43982
O151	O	1.03471	0.27676	0.28171
O152	O	0.90912	0.02799	0.28285
O153	O	0.87851	0.38155	0.28326
O154	O	1.00310	0.13093	0.28145
O155	O	0.90769	0.23698	0.29744
O156	O	1.03255	-0.01327	0.29699
H157	H	0.77139	0.07822	0.60404
H158	H	0.89747	0.33162	0.61184
H159	H	0.98494	0.43747	0.41642
H160	H	0.86108	0.18721	0.41903
Al161	Al	0.12866	0.59266	0.51430
Al162	Al	0.25387	0.84287	0.51444
Si163	Si	0.15181	1.04463	0.69912
Si164	Si	0.02664	0.79461	0.69978
Si165	Si	0.15478	0.70885	0.69984
Si166	Si	0.03025	0.95912	0.69914
O167	O	0.15411	0.52390	0.58692
O168	O	0.02917	0.77415	0.58688
O169	O	0.16975	0.68042	0.58689
O170	O	0.04530	0.93007	0.58659
O171	O	0.04450	0.61803	0.58834
O172	O	0.16982	0.86874	0.58853
O173	O	-0.02672	0.74136	0.74608
O174	O	0.09843	0.99163	0.74561
O175	O	0.12845	0.63640	0.74464
O176	O	0.00417	0.88705	0.74472
O177	O	0.10015	0.78135	0.73018
O178	O	-0.02442	1.03162	0.73063
Al179	Al	0.12933	0.92582	0.51340
Al180	Al	0.00397	0.67579	0.51397
Si181	Si	0.10634	0.47414	0.32799
Si182	Si	0.23149	0.72403	0.32856
Si183	Si	0.10322	0.80941	0.32823
Si184	Si	0.22840	0.55962	0.32897
O185	O	0.10398	0.99425	0.44050
O186	O	0.22907	0.74438	0.44119
O187	O	0.08834	0.83785	0.44111
O188	O	0.21295	0.58851	0.44141
O189	O	0.21355	0.90015	0.43944
O190	O	0.08808	0.65010	0.43956
O191	O	0.28554	0.77647	0.28216
O192	O	0.16007	0.52784	0.28237
O193	O	0.12900	0.88142	0.28274
O194	O	0.25561	0.63179	0.28354
O195	O	0.15816	0.73691	0.29752
O196	O	0.28335	0.48763	0.29743
H197	H	0.02107	0.58206	0.61155

H198	H	0.14736	0.83431	0.61562
H199	H	0.23526	0.93714	0.41589
H200	H	0.11026	0.68537	0.41366
Mg201	Mg	0.37852	0.59296	0.51450
Al202	Al	0.50405	0.84268	0.51504
Si203	Si	0.40182	1.04454	0.70012
Si204	Si	0.27672	0.79441	0.70011
Si205	Si	0.40453	0.70897	0.70055
Si206	Si	0.27978	0.95911	0.69976
O207	O	0.40395	0.52457	0.58702
O208	O	0.27914	0.77429	0.58714
O209	O	0.41958	0.68033	0.58721
O210	O	0.29516	0.93037	0.58694
O211	O	0.29475	0.61811	0.58796
O212	O	0.41992	0.86840	0.58907
O213	O	0.22326	0.74080	0.74586
O214	O	0.34847	0.99078	0.74560
O215	O	0.37870	0.63683	0.74616
O216	O	0.25353	0.88671	0.74460
O217	O	0.35021	0.78173	0.73079
O218	O	0.22525	1.03177	0.72997
Al219	Al	0.37945	0.92578	0.51429
Al220	Al	0.25409	0.67597	0.51435
Si221	Si	0.35661	0.47422	0.32839
Si222	Si	0.48144	0.72410	0.32998
Si223	Si	0.35348	0.80963	0.32895
Si224	Si	0.47822	0.55960	0.32922
O225	O	0.35435	0.99411	0.44106
O226	O	0.47891	0.74440	0.44224
O227	O	0.33828	0.83812	0.44157
O228	O	0.46253	0.58878	0.44174
O229	O	0.46354	0.89987	0.43999
O230	O	0.33823	0.65022	0.43946
O231	O	0.53451	0.77798	0.28435
O232	O	0.40935	0.52891	0.28217
O233	O	0.37974	0.88155	0.28361
O234	O	0.50376	0.63190	0.28396
O235	O	0.40783	0.73640	0.29829
O236	O	0.53290	0.48722	0.29815
H237	H	0.27272	0.58330	0.61486
H238	H	0.39757	0.83297	0.61488
H239	H	0.48636	0.93590	0.41589
H240	H	0.36135	0.68869	0.42120
Al241	Al	0.62875	0.59266	0.51478
Al242	Al	0.75406	0.84286	0.51426
Si243	Si	0.65160	1.04461	0.69974
Si244	Si	0.52657	0.79445	0.70052
Si245	Si	0.65488	0.70903	0.70016
Si246	Si	0.53007	0.95920	0.70011
O247	O	0.65454	0.52412	0.58753
O248	O	0.52951	0.77409	0.58765
O249	O	0.66984	0.68049	0.58726
O250	O	0.54521	0.93016	0.58743
O251	O	0.54465	0.61782	0.58894
O252	O	0.67020	0.86898	0.58922

O253	O	0.47337	0.74073	0.74604
O254	O	0.59864	0.99127	0.74626
O255	O	0.62934	0.63676	0.74568
O256	O	0.50410	0.88693	0.74538
O257	O	0.60026	0.78136	0.73058
O258	O	0.47529	1.03149	0.73085
Al259	Al	0.62936	0.92613	0.51444
Al260	Al	0.50405	0.67584	0.51506
Si261	Si	0.60644	0.47395	0.32876
Si262	Si	0.73150	0.72408	0.32850
Si263	Si	0.60334	0.81001	0.32943
Si264	Si	0.72837	0.55968	0.32882
O265	O	0.60378	0.99471	0.44167
O266	O	0.72870	0.74448	0.44136
O267	O	0.58848	0.83812	0.44215
O268	O	0.71278	0.58862	0.44155
O269	O	0.71346	0.90084	0.44011
O270	O	0.58785	0.64989	0.44000
O271	O	0.78498	0.77750	0.28226
O272	O	0.65971	0.52764	0.28293
O273	O	0.62922	0.88201	0.28384
O274	O	0.75365	0.63182	0.28266
O275	O	0.65804	0.73757	0.29789
O276	O	0.78275	0.48703	0.29789
H277	H	0.52124	0.58242	0.61338
H278	H	0.64773	0.83419	0.61570
H279	H	0.73572	0.93892	0.41932
H280	H	0.61029	0.68535	0.41471
Mg281	Mg	0.87858	0.59275	0.51437
Al282	Al	1.00391	0.84280	0.51397
Si283	Si	0.90234	1.04431	0.69929
Si284	Si	0.77696	0.79432	0.69985
Si285	Si	0.90491	0.70880	0.70013
Si286	Si	0.78027	0.95871	0.69918
O287	O	0.90402	0.52447	0.58725
O288	O	0.77911	0.77428	0.58719
O289	O	0.91974	0.68007	0.58706
O290	O	0.79546	0.92995	0.58697
O291	O	0.79479	0.61826	0.58851
O292	O	0.92008	0.86873	0.58888
O293	O	0.72332	0.74141	0.74643
O294	O	0.84822	0.99210	0.74609
O295	O	0.87968	0.63687	0.74670
O296	O	0.75450	0.88673	0.74507
O297	O	0.85003	0.78101	0.73142
O298	O	0.72562	1.03161	0.72991
Al299	Al	0.87949	0.92579	0.51399
Al300	Al	0.75410	0.67597	0.51430
Si301	Si	0.85638	0.47410	0.32830
Si302	Si	0.98123	0.72369	0.32845
Si303	Si	0.85343	0.80956	0.32884
Si304	Si	0.97793	0.55941	0.32820
O305	O	0.85378	0.99443	0.44167
O306	O	0.97873	0.74443	0.44119
O307	O	0.83836	0.83817	0.44141

O308	O	0.96259	0.58830	0.44136
O309	O	0.96341	0.90051	0.43946
O310	O	0.83802	0.65008	0.44015
O311	O	1.03447	0.77742	0.28260
O312	O	0.90977	0.52695	0.28145
O313	O	0.87967	0.88174	0.28327
O314	O	1.00409	0.63167	0.28313
O315	O	0.90804	0.73742	0.29723
O316	O	1.03291	0.48726	0.29758
H317	H	0.77253	0.58209	0.61264
H318	H	0.89771	0.83233	0.61271
H319	H	0.98588	0.93869	0.41910
H320	H	0.86097	0.68541	0.41527
C321	C	0.64749	0.69550	0.02222
H322	H	0.66466	0.64908	0.04995
C323	C	0.63827	0.79704	-0.03709
C324	C	0.63890	0.86303	-0.07135
H325	H	0.68415	0.88527	-0.09937
C326	C	0.57928	0.90004	-0.06953
H327	H	0.57640	0.95127	-0.09695
C328	C	0.52176	0.87085	-0.03439
H329	H	0.47364	0.89755	-0.03281
N330	N	0.52097	0.80750	0.00099
C331	C	0.57885	0.77579	-0.00327
C332	C	0.75681	0.73928	-0.03949
H333	H	0.77616	0.79708	-0.01188
H334	H	0.77371	0.72242	-0.11454
N335	N	0.79174	0.68447	0.00534
C336	C	0.86203	0.71266	0.02045
H337	H	0.85696	0.77127	0.06710
H338	H	0.88654	0.71609	-0.04766
C339	C	0.89773	0.65636	0.06075
H340	H	0.87467	0.65810	0.13133
H341	H	0.94796	0.67633	0.06835
C342	C	0.89821	0.57331	-0.00061
H343	H	0.92379	0.53389	0.03085
H344	H	0.92632	0.57023	-0.06872
C345	C	0.82839	0.54656	-0.01462
H346	H	0.82897	0.48787	-0.06124
H347	H	0.80185	0.54327	0.05232
C348	C	0.79181	0.60068	-0.05698
H349	H	0.81582	0.60207	-0.12580
H350	H	0.74070	0.58505	-0.06420
C351	C	0.52969	0.68529	0.09606
H352	H	0.50083	0.73697	0.13625
H353	H	0.55458	0.66421	0.14653
C354	C	0.48314	0.62533	0.05186
C355	C	0.43278	0.64553	0.00668
H356	H	0.43004	0.70456	-0.00278
C357	C	0.38769	0.59180	-0.03029
H358	H	0.35005	0.60852	-0.06895
C359	C	0.39206	0.51600	-0.02194
H360	H	0.35805	0.47272	-0.05441
C361	C	0.44205	0.49493	0.02340
H362	H	0.44717	0.43473	0.02773

C363	C	0.48680	0.54943	0.05988
H364	H	0.52685	0.53173	0.09364
N365	N	0.58076	0.71171	0.03446
H366	H	0.76667	0.68654	0.06796
C367	C	0.68373	0.74443	-0.02114
O368	O	0.67281	0.10728	0.14531
H369	H	0.64671	0.05244	0.20099
H370	H	0.63781	0.17587	0.21056
O371	O	0.79958	0.17251	0.94139
H372	H	0.82765	0.19318	1.03178
H373	H	0.72425	0.18539	0.96204
O374	O	0.14139	0.25363	0.92120
H375	H	0.20015	0.24756	0.84940
H376	H	0.16128	0.24689	1.01002
O377	O	0.97427	0.05229	0.10920
H378	H	1.02117	0.09218	0.17468
H379	H	0.93224	0.02331	0.02877
O380	O	0.92835	0.89931	0.11074
H381	H	0.97620	0.96441	0.11163
H382	H	0.92816	0.87371	0.19692
O383	O	0.80639	0.17861	0.12610
H384	H	0.74229	0.13527	0.12795
H385	H	0.79461	0.25977	0.18230
O386	O	0.02532	0.32991	0.90550
H387	H	0.09299	0.32481	0.93308
H388	H	0.00211	0.38623	0.85868
O389	O	0.67484	0.14415	0.90002
H390	H	0.69046	0.06530	0.91530
H391	H	0.61682	0.15349	0.85167
O392	O	0.85926	0.00122	0.03376
H393	H	0.88479	-0.07222	0.05320
H394	H	0.79877	-0.00931	-0.01560
O395	O	0.73912	0.03292	-0.03317
H396	H	0.68732	0.05262	0.04947
H397	H	0.79146	0.09198	-0.04163
Na398	Na	0.77143	0.14683	0.79260
Na399	Na	0.36693	0.61082	0.19553
Na400	Na	0.86658	0.09078	0.18237
Na401	Na	0.04255	0.19816	0.85077

Table S5. Cell parameters and atomic coordinates of the 4x2x1 supercell of MMT with five molecules of 7AI intercalated (in fractional coordinates).

cell_length_a	20.6400
cell_length_b	17.9320
cell_length_c	22.3470
cell_angle_alpha	90.0000
cell_angle_beta	100.4600
cell_angle_gamma	90.0000

atom	symbol	x	y	z
Mg1	Mg	0.12960	0.06814	0.55501
Al2	Al	0.25483	0.31797	0.55482
Si3	Si	0.19121	0.48084	0.67646
Si4	Si	0.06642	0.23032	0.67632
Si5	Si	0.19420	0.14474	0.67681

Si6	Si	0.06950	0.39485	0.67647
O7	O	0.17036	-0.01553	0.60278
O8	O	0.04540	0.23413	0.60259
O9	O	0.18583	0.14023	0.60266
O10	O	0.06121	0.39007	0.60260
O11	O	0.06121	0.07807	0.60351
O12	O	0.18631	0.32800	0.60372
O13	O	0.02237	0.16859	0.70753
O14	O	0.14793	0.41691	0.70653
O15	O	0.17854	0.06327	0.70726
O16	O	0.05354	0.31303	0.70618
O17	O	0.14617	0.21086	0.69694
O18	O	0.02163	0.46061	0.69703
Al19	Al	0.13017	0.40123	0.55478
Al20	Al	0.00498	0.15125	0.55488
Si21	Si	0.06884	-0.01114	0.43307
Si22	Si	0.19377	0.23833	0.43311
Si23	Si	0.06545	0.32401	0.43330
Si24	Si	0.19056	0.07406	0.43316
O25	O	0.08975	0.48511	0.50703
O26	O	0.21477	0.23522	0.50703
O27	O	0.07383	0.32930	0.50711
O28	O	0.19865	0.07919	0.50726
O29	O	0.19875	0.39132	0.50602
O30	O	0.07360	0.14102	0.50619
O31	O	0.23774	0.30167	0.40322
O32	O	0.11288	0.05110	0.40221
O33	O	0.08071	0.40596	0.40301
O34	O	0.20716	0.15579	0.40344
O35	O	0.11404	0.25899	0.41253
O36	O	0.23950	0.00853	0.41315
H37	H	0.04241	0.03661	0.61740
H38	H	0.16835	0.28615	0.61820
H39	H	0.21668	0.43232	0.49034
H40	H	0.09120	0.18158	0.49002
Al41	Al	0.37987	0.06811	0.55552
Al42	Al	0.50501	0.31820	0.55554
Si43	Si	0.44109	0.48067	0.67696
Si44	Si	0.31568	0.23064	0.67656
Si45	Si	0.44416	0.14523	0.67704
Si46	Si	0.31927	0.39531	0.67626
O47	O	0.42048	-0.01585	0.60333
O48	O	0.29532	0.23411	0.60266
O49	O	0.43582	0.14074	0.60311
O50	O	0.31102	0.39001	0.60255
O51	O	0.31098	0.07803	0.60389
O52	O	0.43624	0.32831	0.60418
O53	O	0.27215	0.16796	0.70695
O54	O	0.39743	0.41707	0.70674
O55	O	0.42773	0.06354	0.70670
O56	O	0.30231	0.31359	0.70609
O57	O	0.39560	0.21111	0.69711
O58	O	0.27099	0.46110	0.69640
Al59	Al	0.38022	0.40141	0.55497
Al60	Al	0.25494	0.15120	0.55488

Si61	Si	0.31919	-0.01120	0.43356
Si62	Si	0.44426	0.23867	0.43362
Si63	Si	0.31588	0.32431	0.43339
Si64	Si	0.44133	0.07434	0.43401
O65	O	0.33985	0.48543	0.50736
O66	O	0.46482	0.23530	0.50763
O67	O	0.32424	0.32897	0.50734
O68	O	0.44899	0.07940	0.50781
O69	O	0.44911	0.39154	0.50663
O70	O	0.32373	0.14104	0.50630
O71	O	0.48800	0.30189	0.40362
O72	O	0.36338	0.05160	0.40344
O73	O	0.33230	0.40597	0.40366
O74	O	0.45768	0.15598	0.40384
O75	O	0.36441	0.25844	0.41336
O76	O	0.48953	0.00843	0.41373
H77	H	0.29217	0.03727	0.61899
H78	H	0.41869	0.28739	0.62010
H79	H	0.46617	0.43336	0.49140
H80	H	0.34050	0.18142	0.48898
Mg81	Mg	0.62976	0.06818	0.55624
Al82	Al	0.75519	0.31772	0.55589
Si83	Si	0.69182	0.48059	0.67766
Si84	Si	0.56644	0.23043	0.67741
Si85	Si	0.69481	0.14483	0.67794
Si86	Si	0.56955	0.39509	0.67717
O87	O	0.66985	-0.01552	0.60404
O88	O	0.54512	0.23433	0.60348
O89	O	0.68597	0.14020	0.60380
O90	O	0.56133	0.39022	0.60331
O91	O	0.56104	0.07833	0.60441
O92	O	0.68645	0.32786	0.60457
O93	O	0.52186	0.16847	0.70801
O94	O	0.64748	0.41772	0.70770
O95	O	0.67928	0.06328	0.70850
O96	O	0.55317	0.31333	0.70709
O97	O	0.64612	0.21046	0.69824
O98	O	0.52085	0.46040	0.69754
Al99	Al	0.63058	0.40120	0.55567
Al100	Al	0.50518	0.15145	0.55557
Si101	Si	0.56926	-0.01146	0.43440
Si102	Si	0.69386	0.23815	0.43432
Si103	Si	0.56603	0.32443	0.43395
Si104	Si	0.69090	0.07415	0.43486
O105	O	0.59022	0.48517	0.50793
O106	O	0.71480	0.23504	0.50809
O107	O	0.57424	0.32926	0.50788
O108	O	0.69849	0.07923	0.50845
O109	O	0.69914	0.39111	0.50695
O110	O	0.57388	0.14103	0.50710
O111	O	0.73777	0.30083	0.40363
O112	O	0.61304	0.05121	0.40364
O113	O	0.58260	0.40616	0.40434
O114	O	0.70613	0.15565	0.40410
O115	O	0.61422	0.25866	0.41369

O116	O	0.73923	0.00932	0.41320
H117	H	0.54424	0.03746	0.62102
H118	H	0.66913	0.28626	0.61993
H119	H	0.71729	0.43224	0.49170
H120	H	0.59236	0.18200	0.49219
Mg121	Mg	0.87962	0.06825	0.55527
Al122	Al	1.00476	0.31787	0.55507
Si123	Si	0.94176	0.48087	0.67734
Si124	Si	0.81662	0.23016	0.67737
Si125	Si	0.94472	0.14480	0.67662
Si126	Si	0.82017	0.39499	0.67729
O127	O	0.92036	-0.01542	0.60283
O128	O	0.79554	0.23394	0.60360
O129	O	0.93586	0.14049	0.60270
O130	O	0.81135	0.39001	0.60338
O131	O	0.81155	0.07784	0.60420
O132	O	0.93651	0.32785	0.60416
O133	O	0.77267	0.16816	0.70844
O134	O	0.89831	0.41726	0.70739
O135	O	0.92973	0.06329	0.70724
O136	O	0.80417	0.31310	0.70718
O137	O	0.89645	0.21011	0.69794
O138	O	0.77149	0.46020	0.69802
Al139	Al	0.88036	0.40121	0.55552
Al140	Al	0.75518	0.15111	0.55594
Si141	Si	0.81888	-0.01110	0.43370
Si142	Si	0.94358	0.23815	0.43348
Si143	Si	0.81560	0.32414	0.43408
Si144	Si	0.94058	0.07405	0.43341
O145	O	0.83999	0.48522	0.50767
O146	O	0.96467	0.23520	0.50714
O147	O	0.82415	0.32902	0.50788
O148	O	0.94818	0.07965	0.50722
O149	O	0.94868	0.39138	0.50641
O150	O	0.82322	0.14101	0.50663
O151	O	0.98742	0.30107	0.40284
O152	O	0.86271	0.05081	0.40234
O153	O	0.83148	0.40568	0.40388
O154	O	0.95564	0.15588	0.40284
O155	O	0.86402	0.25919	0.41272
O156	O	0.98912	0.00888	0.41261
H157	H	0.79288	0.03631	0.61810
H158	H	0.91868	0.28622	0.61895
H159	H	0.96606	0.43379	0.49249
H160	H	0.84137	0.18216	0.49178
Al161	Al	0.12970	0.56813	0.55490
Al162	Al	0.25496	0.81811	0.55520
Si163	Si	0.19122	0.98089	0.67697
Si164	Si	0.06622	0.73086	0.67659
Si165	Si	0.19429	0.64504	0.67653
Si166	Si	0.06961	0.89535	0.67638
O167	O	0.17024	0.48415	0.60260
O168	O	0.04538	0.73428	0.60267
O169	O	0.18598	0.64030	0.60263
O170	O	0.06135	0.88994	0.60270

O171	O	0.06120	0.57810	0.60366
O172	O	0.18633	0.82811	0.60384
O173	O	0.02293	0.66779	0.70697
O174	O	0.14763	0.91730	0.70687
O175	O	0.17780	0.56334	0.70616
O176	O	0.05310	0.81361	0.70641
O177	O	0.14612	0.71106	0.69660
O178	O	0.02130	0.96045	0.69721
Al179	Al	0.13027	0.90145	0.55491
Al180	Al	0.00499	0.65141	0.55496
Si181	Si	0.06872	0.48884	0.43319
Si182	Si	0.19399	0.73876	0.43316
Si183	Si	0.06556	0.82452	0.43335
Si184	Si	0.19102	0.57427	0.43328
O185	O	0.09017	0.98531	0.50706
O186	O	0.21481	0.73524	0.50717
O187	O	0.07418	0.82896	0.50731
O188	O	0.19885	0.57926	0.50720
O189	O	0.19907	0.89144	0.50634
O190	O	0.07349	0.64129	0.50600
O191	O	0.23809	0.80178	0.40309
O192	O	0.11290	0.55137	0.40288
O193	O	0.08170	0.90604	0.40337
O194	O	0.20685	0.65610	0.40330
O195	O	0.11415	0.75882	0.41307
O196	O	0.23926	0.50849	0.41299
H197	H	0.04247	0.53746	0.61899
H198	H	0.16899	0.78776	0.62070
H199	H	0.21600	0.93353	0.49143
H200	H	0.09043	0.68200	0.48922
Mg201	Mg	0.37977	0.56814	0.55536
Al202	Al	0.50523	0.81792	0.55612
Si203	Si	0.44127	0.98075	0.67736
Si204	Si	0.31625	0.73036	0.67688
Si205	Si	0.44430	0.64474	0.67728
Si206	Si	0.31927	0.89495	0.67698
O207	O	0.42003	0.48437	0.60302
O208	O	0.29524	0.73412	0.60295
O209	O	0.43602	0.63987	0.60329
O210	O	0.31130	0.89005	0.60303
O211	O	0.31126	0.57808	0.60382
O212	O	0.43633	0.82794	0.60448
O213	O	0.27253	0.66694	0.70703
O214	O	0.39743	0.91733	0.70720
O215	O	0.42797	0.56303	0.70742
O216	O	0.30294	0.81298	0.70658
O217	O	0.39597	0.71042	0.69746
O218	O	0.27103	0.96067	0.69713
Al219	Al	0.38049	0.90123	0.55546
Al220	Al	0.25515	0.65133	0.55495
Si221	Si	0.31913	0.48883	0.43318
Si222	Si	0.44422	0.73832	0.43433
Si223	Si	0.31600	0.82434	0.43366
Si224	Si	0.44086	0.57418	0.43383
O225	O	0.34031	0.98509	0.50749

O226	O	0.46508	0.73520	0.50811
O227	O	0.32435	0.82914	0.50758
O228	O	0.44886	0.57924	0.50788
O229	O	0.44936	0.89121	0.50692
O230	O	0.32405	0.64123	0.50671
O231	O	0.48821	0.80131	0.40409
O232	O	0.36307	0.55162	0.40282
O233	O	0.33269	0.90604	0.40396
O234	O	0.45733	0.65607	0.40412
O235	O	0.36473	0.75916	0.41311
O236	O	0.48948	0.50861	0.41376
H237	H	0.29376	0.53673	0.61913
H238	H	0.41882	0.78674	0.62022
H239	H	0.46783	0.93276	0.49260
H240	H	0.34173	0.68182	0.49053
Al241	Al	0.63012	0.56809	0.55594
Al242	Al	0.75519	0.81843	0.55566
Si243	Si	0.69174	0.98089	0.67804
Si244	Si	0.56635	0.73052	0.67771
Si245	Si	0.69499	0.64516	0.67757
Si246	Si	0.56984	0.89532	0.67771
O247	O	0.67062	0.48404	0.60365
O248	O	0.54548	0.73388	0.60375
O249	O	0.68622	0.64058	0.60353
O250	O	0.56137	0.88994	0.60380
O251	O	0.56124	0.57774	0.60447
O252	O	0.68686	0.82833	0.60479
O253	O	0.52228	0.66767	0.70812
O254	O	0.64792	0.91765	0.70818
O255	O	0.67851	0.56331	0.70718
O256	O	0.55317	0.81338	0.70723
O257	O	0.64618	0.71060	0.69782
O258	O	0.52103	0.96080	0.69792
Al259	Al	0.63050	0.90147	0.55614
Al260	Al	0.50530	0.65114	0.55574
Si261	Si	0.56922	0.48883	0.43395
Si262	Si	0.69415	0.73866	0.43408
Si263	Si	0.56621	0.82427	0.43470
Si264	Si	0.69111	0.57446	0.43412
O265	O	0.58965	0.98525	0.50837
O266	O	0.71474	0.73537	0.50795
O267	O	0.57450	0.82889	0.50850
O268	O	0.69900	0.57951	0.50801
O269	O	0.69883	0.89181	0.50706
O270	O	0.57390	0.64128	0.50700
O271	O	0.73743	0.80198	0.40372
O272	O	0.61290	0.55225	0.40404
O273	O	0.58177	0.90581	0.40444
O274	O	0.70689	0.65621	0.40405
O275	O	0.61433	0.75873	0.41400
O276	O	0.73904	0.50852	0.41358
H277	H	0.54259	0.53671	0.61923
H278	H	0.67025	0.78812	0.62244
H279	H	0.71711	0.93454	0.49436
H280	H	0.59127	0.68200	0.49066

Mg281	Mg	0.87987	0.56808	0.55548
Al282	Al	1.00481	0.81835	0.55499
Si283	Si	0.94185	0.98109	0.67658
Si284	Si	0.81703	0.73075	0.67727
Si285	Si	0.94481	0.64489	0.67715
Si286	Si	0.81995	0.89552	0.67678
O287	O	0.92050	0.48438	0.60315
O288	O	0.79559	0.73444	0.60337
O289	O	0.93612	0.64008	0.60297
O290	O	0.81157	0.89025	0.60312
O291	O	0.81156	0.57810	0.60407
O292	O	0.93655	0.82837	0.60412
O293	O	0.77292	0.66822	0.70785
O294	O	0.89819	0.91792	0.70721
O295	O	0.92916	0.56334	0.70753
O296	O	0.80436	0.81370	0.70692
O297	O	0.89685	0.71105	0.69741
O298	O	0.77159	0.96050	0.69802
Al299	Al	0.88042	0.90160	0.55516
Al300	Al	0.75539	0.65149	0.55570
Si301	Si	0.81878	0.48857	0.43365
Si302	Si	0.94339	0.73872	0.43331
Si303	Si	0.81551	0.82477	0.43376
Si304	Si	0.94024	0.57442	0.43346
O305	O	0.83994	0.98538	0.50746
O306	O	0.96456	0.73538	0.50722
O307	O	0.82412	0.82943	0.50761
O308	O	0.94864	0.57914	0.50747
O309	O	0.94878	0.89163	0.50620
O310	O	0.82379	0.64105	0.50685
O311	O	0.98755	0.80142	0.40302
O312	O	0.86275	0.55058	0.40246
O313	O	0.83063	0.90635	0.40329
O314	O	0.95615	0.65603	0.40337
O315	O	0.86373	0.75941	0.41282
O316	O	0.98908	0.50924	0.41275
H317	H	0.79486	0.53733	0.62092
H318	H	0.91869	0.78686	0.61901
H319	H	0.96675	0.93414	0.49279
H320	H	0.84162	0.68182	0.49100
C321	C	0.35882	0.23288	0.87069
H322	H	0.33147	0.28486	0.87262
C323	C	0.43146	0.14563	0.85607
C324	C	0.47939	0.09586	0.84307
H325	H	0.52156	0.11425	0.82308
C326	C	0.47009	0.02032	0.85336
H327	H	0.50483	-0.02162	0.84189
C328	C	0.41520	-0.00291	0.87715
H329	H	0.40580	-0.06202	0.88524
N330	N	0.36876	0.04544	0.89118
C331	C	0.37990	0.11535	0.87849
C332	C	0.45545	0.28641	0.83008
H333	H	0.43086	0.30570	0.78465
H334	H	0.50514	0.26803	0.82511
N335	N	0.46478	0.35599	0.87053

C336	C	0.50779	0.41281	0.84258
H337	H	0.48032	0.42656	0.79706
H338	H	0.55378	0.38409	0.83760
C339	C	0.52207	0.48135	0.88326
H340	H	0.47661	0.51428	0.88177
H341	H	0.55649	0.51712	0.86373
C342	C	0.55122	0.46077	0.94941
H343	H	0.55968	0.51190	0.97735
H344	H	0.60005	0.43460	0.95154
C345	C	0.50508	0.40734	0.97528
H346	H	0.52589	0.39176	1.02266
H347	H	0.45769	0.43535	0.97655
C348	C	0.49274	0.33606	0.93754
H349	H	0.53869	0.30446	0.93806
H350	H	0.45659	0.29937	0.95350
C351	C	0.26721	0.15511	0.90676
H352	H	0.26374	0.09621	0.91964
H353	H	0.22885	0.16249	0.86596
C354	C	0.25339	0.20451	0.95793
C355	C	0.28257	0.19043	1.01829
H356	H	0.31637	0.14278	1.02833
C357	C	0.26953	0.23513	1.06573
H358	H	0.29293	0.22275	1.11292
C359	C	0.22669	0.29580	1.05327
H360	H	0.21638	0.33151	1.09042
C361	C	0.19747	0.31091	0.99304
H362	H	0.16411	0.35883	0.98230
C363	C	0.21089	0.26543	0.94622
H364	H	0.18785	0.27805	0.89890
N365	N	0.33213	0.16731	0.88879
H366	H	0.41958	0.37988	0.86895
C367	C	0.41825	0.22268	0.85113
C368	C	0.65496	0.91854	0.27506
H369	H	0.69654	0.90405	0.31116
C370	C	0.58157	0.97754	0.20546
C371	C	0.54087	1.02072	0.16172
H372	H	0.54664	1.08143	0.15893
C373	C	0.49246	0.98363	0.12099
H374	H	0.45926	1.01428	0.08517
C375	C	0.48510	0.90644	0.12585
H376	H	0.44760	0.87572	0.09398
N377	N	0.52378	0.86399	0.16941
C378	C	0.56961	0.90251	0.20494
C379	C	0.67714	1.05652	0.27121
H380	H	0.67620	1.06861	0.31916
H381	H	0.65572	1.10575	0.24542
N382	N	0.74988	1.05383	0.26524
C383	C	0.78288	1.12868	0.28961
H384	H	0.78234	1.13039	0.33877
H385	H	0.75148	1.17429	0.26749
C386	C	0.85237	1.13389	0.27531
H387	H	0.88469	1.09234	0.30229
H388	H	0.87265	1.18900	0.29067
C389	C	0.85338	1.12128	0.20751
H390	H	0.90420	1.12645	0.19885

H391	H	0.82420	1.16470	0.17966
C392	C	0.82569	1.04364	0.18852
H393	H	0.82636	1.03277	0.14001
H394	H	0.85695	1.00040	0.21457
C395	C	0.75480	1.03577	0.19839
H396	H	0.72199	1.07502	0.16924
H397	H	0.73561	0.97874	0.18907
C398	C	0.62697	0.78017	0.25184
H399	H	0.58152	0.74991	0.23305
H400	H	0.63848	0.76428	0.30018
C401	C	0.68190	0.75618	0.21948
C402	C	0.67099	0.74855	0.15623
H403	H	0.62137	0.75905	0.13007
C404	C	0.72151	0.72805	0.12580
H405	H	0.71150	0.72216	0.07597
C406	C	0.78485	0.71506	0.15869
H407	H	0.82505	0.69909	0.13509
C408	C	0.79652	0.72242	0.22201
H409	H	0.84599	0.71244	0.24885
C410	C	0.74530	0.74254	0.25165
H411	H	0.75519	0.74737	0.30160
N412	N	0.61423	0.86161	0.24788
H413	H	0.77184	1.01232	0.29257
C414	C	0.63748	0.98843	0.25095
C415	C	0.10934	0.98365	0.85469
H416	H	0.09766	0.96447	0.80740
C417	C	0.10681	1.03524	0.94504
C418	C	0.09851	1.06922	0.99959
H419	H	0.05327	1.09925	1.00385
C420	C	0.15037	1.06413	1.04885
H421	H	0.14691	1.09035	1.09272
C422	C	0.20791	1.02615	1.04234
H423	H	0.25006	1.02149	1.08001
N424	N	0.21650	0.99266	0.98921
C425	C	0.16540	0.99966	0.94492
C426	C	0.00109	1.04709	0.85849
H427	H	-0.00234	1.05854	0.80958
H428	H	-0.01248	1.09908	0.87942
N429	N	-0.05306	0.99041	0.86364
C430	C	-0.12061	1.02283	0.83216
H431	H	-0.11839	1.03026	0.78386
H432	H	-0.12571	1.07822	0.85221
C433	C	-0.17648	0.97078	0.84143
H434	H	-0.17355	0.91849	0.81598
H435	H	-0.22349	0.99750	0.82088
C436	C	-0.17476	0.95287	0.90890
H437	H	-0.21570	0.91488	0.91363
H438	H	-0.18263	1.00430	0.93381
C439	C	-0.10835	0.91742	0.93665
H440	H	-0.10591	0.90473	0.98534
H441	H	-0.10263	0.86375	0.91391
C442	C	-0.05069	0.96859	0.93092
H443	H	-0.05254	1.02069	0.95680
H444	H	-0.00283	0.94119	0.94665
C445	C	0.22405	0.91984	0.87211

H446	H	0.27186	0.94180	0.89530
H447	H	0.22418	0.92774	0.82324
C448	C	0.21837	0.83789	0.88639
C449	C	0.23969	0.81048	0.94518
H450	H	0.26189	0.84934	0.98119
C451	C	0.23351	0.73529	0.95887
H452	H	0.25119	0.71484	1.00533
C453	C	0.20541	0.68562	0.91326
H454	H	0.20055	0.62611	0.92343
C455	C	0.18410	0.71216	0.85417
H456	H	0.16203	0.67364	0.81778
C457	C	0.19076	0.78751	0.84131
H458	H	0.17419	0.80776	0.79453
N459	N	0.17052	0.96601	0.88909
H460	H	-0.04354	0.94364	0.84069
C461	C	0.06968	1.02461	0.88634
C462	C	0.47077	0.69058	0.16417
H463	H	0.49017	0.74137	0.14701
C464	C	0.42911	0.60501	0.21886
C465	C	0.40317	0.55639	0.25749
H466	H	0.38978	0.57515	0.30039
C467	C	0.39531	0.48190	0.24004
H468	H	0.37592	0.44114	0.26919
C469	C	0.41268	0.45858	0.18529
H470	H	0.40720	0.40042	0.16984
N471	N	0.43785	0.50579	0.14712
C472	C	0.44419	0.57480	0.16688
C473	C	0.44381	0.74315	0.26200
H474	H	0.49276	0.75088	0.29103
H475	H	0.40957	0.72931	0.29290
N476	N	0.42296	0.81987	0.23495
C477	C	0.43136	0.87817	0.28750
H478	H	0.48420	0.87948	0.30762
H479	H	0.40353	0.85730	0.32177
C480	C	0.40516	0.95396	0.26314
H481	H	0.43633	0.97705	0.23251
H482	H	0.40962	0.99273	0.30207
C483	C	0.33318	0.94897	0.23007
H484	H	0.31545	1.00448	0.21285
H485	H	0.30158	0.93238	0.26284
C486	C	0.32579	0.89233	0.17763
H487	H	0.27354	0.88655	0.15583
H488	H	0.35198	0.91245	0.14194
C489	C	0.35093	0.81521	0.19979
H490	H	0.32096	0.79183	0.23150
H491	H	0.35057	0.77546	0.16210
C492	C	0.49667	0.61343	0.07307
H493	H	0.49157	0.55384	0.06110
H494	H	0.55019	0.62383	0.08273
C495	C	0.46439	0.66036	0.01954
C496	C	0.41180	0.63248	-0.02274
H497	H	0.39362	0.57570	-0.01707
C498	C	0.38195	0.67492	-0.07236
H499	H	0.34081	0.65157	-0.10530
C500	C	0.40407	0.74722	-0.08043

H501	H	0.38003	0.78072	-0.11945
C502	C	0.45665	0.77598	-0.03849
H503	H	0.47487	0.83257	-0.04464
C504	C	0.48631	0.73255	0.01065
H505	H	0.52817	0.75548	0.04285
N506	N	0.47064	0.62579	0.13002
H507	H	0.45365	0.83396	0.20588
C508	C	0.44665	0.68082	0.21770
C509	C	1.01410	0.33023	0.15818
H510	H	1.00680	0.38221	0.13216
C511	C	1.02450	0.24309	0.23063
C512	C	1.02889	0.19301	0.27927
H513	H	1.02178	0.20997	0.32482
C514	C	1.04395	0.11904	0.26867
H515	H	1.04849	0.07760	0.30552
C516	C	1.05408	0.09717	0.21102
H517	H	1.06656	0.03945	0.20124
N518	N	1.04917	0.14545	0.16295
C519	C	1.03532	0.21416	0.17667
C520	C	0.99194	0.38086	0.25784
H521	H	1.02763	0.42713	0.25971
H522	H	0.99388	0.36235	0.30504
N523	N	0.92378	0.41492	0.23675
C524	C	0.91491	0.48457	0.27587
H525	H	0.95587	0.52294	0.27266
H526	H	0.91950	0.46516	0.32325
C527	C	0.84720	0.51894	0.25308
H528	H	0.84490	0.54135	0.20679
H529	H	0.84115	0.56704	0.28245
C530	C	0.79135	0.46219	0.25354
H531	H	0.74329	0.48789	0.23469
H532	H	0.78873	0.44745	0.30100
C533	C	0.80159	0.39095	0.21805
H534	H	0.76382	0.34898	0.22312
H535	H	0.79532	0.40359	0.16903
C536	C	0.86939	0.35632	0.23998
H537	H	0.87529	0.33784	0.28780
H538	H	0.87894	0.30861	0.21175
C539	C	1.03378	0.25405	0.06387
H540	H	1.07313	0.21290	0.06183
H541	H	1.05140	0.30642	0.04664
C542	C	0.97039	0.22927	0.02239
C543	C	0.92517	0.18062	0.04146
H544	H	0.93587	0.15743	0.08778
C545	C	0.86691	0.16011	0.00308
H546	H	0.83210	0.12217	0.01951
C547	C	0.85266	0.18774	-0.05630
H548	H	0.80682	0.17213	-0.08712
C549	C	0.89792	0.23494	-0.07665
H550	H	0.88815	0.25606	-0.12375
C551	C	0.95593	0.25486	-0.03768
H552	H	0.99130	0.29129	-0.05514
N553	N	1.02938	0.26594	0.12884
H554	H	0.92015	0.43132	0.19257
C555	C	1.01017	0.31902	0.21893

O556	O	0.23409	0.35613	0.79831
H557	H	0.24813	0.32307	0.76819
H558	H	0.19340	0.37638	0.77419
O559	O	0.35059	0.43300	0.85614
H560	H	0.30603	0.41335	0.84056
H561	H	0.34626	0.48292	0.83697
O562	O	0.35007	0.99235	0.00232
H563	H	0.35736	1.01209	-0.03704
H564	H	0.30165	0.98944	-0.00579
O565	O	0.85723	0.36954	0.01282
H566	H	0.87504	0.32099	0.00390
H567	H	0.83947	0.38707	-0.02825
O568	O	0.89832	0.45973	0.11664
H569	H	0.89639	0.50643	0.09403
H570	H	0.88266	0.42495	0.08272
O571	O	0.54554	0.12293	0.30827
H572	H	0.51433	0.15486	0.32436
H573	H	0.56902	0.10138	0.34657
O574	O	0.34750	0.56973	0.78972
H575	H	0.37700	0.56578	0.75999
H576	H	0.31428	0.60383	0.76779
O577	O	0.97521	0.86367	0.80527
H578	H	1.00644	0.82816	0.79306
H579	H	0.94448	0.86867	0.76625
O580	O	0.98242	0.21653	0.81022
H581	H	1.01505	0.20551	0.78461
H582	H	0.94770	0.23816	0.77935
O583	O	0.53318	0.65974	0.83405
H584	H	0.53721	0.66264	0.79116
H585	H	0.49117	0.68414	0.83426

Table S6. Cell parameters and atomic coordinates of the 4x2x1 supercell of MMT with five molecules of 5OMe-7AI intercalated (in fractional coordinates).

cell_length_a	20.6618
cell_length_b	17.8976
cell_length_c	19.2761
cell_angle_alpha	90.7442
cell_angle_beta	105.7931
cell_angle_gamma	90.1246

atom	symbol	x	y	z
Mg1	Mg	0.13658	0.06987	0.50954
Al2	Al	0.26200	0.31970	0.51009
Si3	Si	0.21104	0.48446	0.65419
Si4	Si	0.08573	0.23398	0.65375
Si5	Si	0.21383	0.14874	0.65403
Si6	Si	0.08905	0.39871	0.65384
O7	O	0.18233	-0.01304	0.56583
O8	O	0.05724	0.23655	0.56626
O9	O	0.19783	0.14273	0.56617
O10	O	0.07318	0.39263	0.56629
O11	O	0.07324	0.08016	0.56693
O12	O	0.19842	0.33032	0.56768
O13	O	0.04493	0.17194	0.69003
O14	O	0.17032	0.42161	0.68965

O15	O	0.20130	0.06736	0.68982
O16	O	0.07570	0.31734	0.68897
O17	O	0.16765	0.21475	0.67816
O18	O	0.04303	0.46505	0.67773
Al19	Al	0.13726	0.40274	0.50984
Al20	Al	0.01201	0.15280	0.50945
Si21	Si	0.06306	-0.01155	0.36502
Si22	Si	0.18807	0.23789	0.36574
Si23	Si	0.05984	0.32343	0.36587
Si24	Si	0.18484	0.07375	0.36533
O25	O	0.09194	0.48580	0.45309
O26	O	0.21708	0.23602	0.45316
O27	O	0.07602	0.32976	0.45331
O28	O	0.20058	0.08015	0.45302
O29	O	0.20076	0.39208	0.45198
O30	O	0.07559	0.14195	0.45172
O31	O	0.22872	0.30064	0.32984
O32	O	0.10398	0.04993	0.32792
O33	O	0.07322	0.40481	0.33058
O34	O	0.19812	0.15501	0.33004
O35	O	0.10634	0.25804	0.34118
O36	O	0.23164	0.00802	0.34147
H37	H	0.05542	0.03864	0.58254
H38	H	0.18263	0.28922	0.58643
H39	H	0.21756	0.43349	0.43483
H40	H	0.09164	0.18285	0.43343
Al41	Al	0.38688	0.06968	0.51028
Al42	Al	0.51175	0.31991	0.50961
Si43	Si	0.46121	0.48472	0.65397
Si44	Si	0.33595	0.23440	0.65418
Si45	Si	0.46438	0.14896	0.65427
Si46	Si	0.33937	0.39931	0.65400
O47	O	0.43254	-0.01347	0.56678
O48	O	0.30766	0.23659	0.56645
O49	O	0.44805	0.14295	0.56658
O50	O	0.32316	0.39285	0.56639
O51	O	0.32312	0.08020	0.56766
O52	O	0.44850	0.33094	0.56763
O53	O	0.29516	0.17177	0.68976
O54	O	0.42067	0.42211	0.68984
O55	O	0.45114	0.06756	0.68946
O56	O	0.32602	0.31786	0.68914
O57	O	0.41796	0.21501	0.67813
O58	O	0.29299	0.46528	0.67808
Al59	Al	0.38717	0.40322	0.50974
Al60	Al	0.26212	0.15283	0.50986
Si61	Si	0.31324	-0.01174	0.36633
Si62	Si	0.43799	0.23817	0.36537
Si63	Si	0.30996	0.32388	0.36583
Si64	Si	0.43540	0.07367	0.36594
O65	O	0.34156	0.48623	0.45307
O66	O	0.46627	0.23601	0.45307
O67	O	0.32602	0.32997	0.45336
O68	O	0.45077	0.07994	0.45345
O69	O	0.45053	0.39250	0.45190

O70	O	0.32556	0.14181	0.45200
O71	O	0.47862	0.30083	0.32964
O72	O	0.35428	0.04995	0.32998
O73	O	0.32227	0.40502	0.32990
O74	O	0.44728	0.15492	0.32986
O75	O	0.35607	0.25783	0.34159
O76	O	0.48120	0.00762	0.34117
H77	H	0.30592	0.03964	0.58560
H78	H	0.43347	0.29095	0.58838
H79	H	0.46606	0.43417	0.43388
H80	H	0.34108	0.18240	0.43253
Mg81	Mg	0.63648	0.06997	0.50966
Al82	Al	0.76193	0.31949	0.50962
Si83	Si	0.71130	0.48445	0.65373
Si84	Si	0.58627	0.23449	0.65379
Si85	Si	0.71429	0.14898	0.65378
Si86	Si	0.58920	0.39901	0.65358
O87	O	0.68145	-0.01291	0.56645
O88	O	0.55697	0.23691	0.56633
O89	O	0.69787	0.14267	0.56612
O90	O	0.57326	0.39284	0.56603
O91	O	0.57329	0.08077	0.56744
O92	O	0.69844	0.33050	0.56745
O93	O	0.54574	0.17189	0.69014
O94	O	0.67039	0.42214	0.68939
O95	O	0.70121	0.06787	0.69002
O96	O	0.57576	0.31770	0.68867
O97	O	0.66803	0.21513	0.67773
O98	O	0.54310	0.46531	0.67744
Al99	Al	0.63732	0.40282	0.50939
Al100	Al	0.51203	0.15308	0.50977
Si101	Si	0.56325	-0.01167	0.36532
Si102	Si	0.68791	0.23769	0.36548
Si103	Si	0.55992	0.32364	0.36530
Si104	Si	0.68499	0.07356	0.36530
O105	O	0.59175	0.48585	0.45290
O106	O	0.71658	0.23587	0.45293
O107	O	0.57586	0.32994	0.45293
O108	O	0.70020	0.08004	0.45293
O109	O	0.70075	0.39206	0.45170
O110	O	0.57533	0.14217	0.45211
O111	O	0.72871	0.30027	0.32970
O112	O	0.60395	0.05003	0.32861
O113	O	0.57314	0.40503	0.33013
O114	O	0.69749	0.15488	0.32940
O115	O	0.60609	0.25775	0.34109
O116	O	0.73137	0.00819	0.34037
H117	H	0.55789	0.03968	0.58618
H118	H	0.68277	0.28909	0.58579
H119	H	0.71702	0.43273	0.43286
H120	H	0.59170	0.18255	0.43317
Mg121	Mg	0.88641	0.06996	0.50915
Al122	Al	1.01174	0.31956	0.50987
Si123	Si	0.96121	0.48474	0.65375
Si124	Si	0.83604	0.23393	0.65355

Si125	Si	0.96391	0.14854	0.65346
Si126	Si	0.83948	0.39888	0.65345
O127	O	0.93181	-0.01299	0.56532
O128	O	0.80725	0.23652	0.56614
O129	O	0.94781	0.14263	0.56587
O130	O	0.82307	0.39281	0.56601
O131	O	0.82330	0.08035	0.56688
O132	O	0.94831	0.33066	0.56764
O133	O	0.79520	0.17237	0.69013
O134	O	0.92050	0.42228	0.68963
O135	O	0.95223	0.06716	0.68965
O136	O	0.82640	0.31751	0.68873
O137	O	0.91783	0.21427	0.67822
O138	O	0.79330	0.46504	0.67808
Al139	Al	0.88714	0.40294	0.50943
Al140	Al	0.76200	0.15278	0.50940
Si141	Si	0.81316	-0.01148	0.36503
Si142	Si	0.93790	0.23796	0.36555
Si143	Si	0.80988	0.32368	0.36569
Si144	Si	0.93512	0.07366	0.36541
O145	O	0.84164	0.48613	0.45287
O146	O	0.96680	0.23595	0.45290
O147	O	0.82599	0.32987	0.45307
O148	O	0.95041	0.08025	0.45278
O149	O	0.95079	0.39221	0.45189
O150	O	0.82531	0.14211	0.45150
O151	O	0.97884	0.30029	0.32961
O152	O	0.85423	0.05038	0.32870
O153	O	0.82267	0.40474	0.33002
O154	O	0.94731	0.15507	0.32917
O155	O	0.85615	0.25801	0.34110
O156	O	0.98148	0.00826	0.34051
H157	H	0.80601	0.03893	0.58319
H158	H	0.93214	0.28928	0.58550
H159	H	0.96621	0.43403	0.43409
H160	H	0.84171	0.18304	0.43358
Al161	Al	0.13671	0.56973	0.50986
Al162	Al	0.26178	0.81975	0.50987
Si163	Si	0.21079	0.98460	0.65360
Si164	Si	0.08572	0.73462	0.65302
Si165	Si	0.21407	0.64892	0.65387
Si166	Si	0.08862	0.89912	0.65203
O167	O	0.18228	0.48662	0.56649
O168	O	0.05719	0.73681	0.56542
O169	O	0.19798	0.64290	0.56627
O170	O	0.07304	0.89258	0.56513
O171	O	0.07292	0.58064	0.56716
O172	O	0.19817	0.83067	0.56734
O173	O	0.04516	0.67243	0.68916
O174	O	0.16973	0.92196	0.68894
O175	O	0.20055	0.56771	0.68909
O176	O	0.07590	0.81801	0.68794
O177	O	0.16770	0.71530	0.67728
O178	O	0.04232	0.96437	0.67686
Al179	Al	0.13718	0.90305	0.50900

Al180	Al	0.01181	0.65297	0.50891
Si181	Si	0.06292	0.48814	0.36558
Si182	Si	0.18793	0.73820	0.36543
Si183	Si	0.05960	0.82401	0.36494
Si184	Si	0.18520	0.57370	0.36582
O185	O	0.09238	0.98618	0.45245
O186	O	0.21645	0.73599	0.45307
O187	O	0.07603	0.82978	0.45247
O188	O	0.20078	0.57986	0.45328
O189	O	0.20104	0.89229	0.45170
O190	O	0.07558	0.64191	0.45146
O191	O	0.22891	0.80079	0.32986
O192	O	0.10419	0.54997	0.32943
O193	O	0.07274	0.90527	0.32948
O194	O	0.19765	0.65504	0.33004
O195	O	0.10617	0.75804	0.34104
O196	O	0.23116	0.50770	0.34129
H197	H	0.05571	0.53997	0.58495
H198	H	0.18244	0.79039	0.58705
H199	H	0.21676	0.93444	0.43476
H200	H	0.09057	0.68226	0.43118
Mg201	Mg	0.38653	0.56994	0.50960
Al202	Al	0.51187	0.81971	0.50968
Si203	Si	0.46115	0.98435	0.65440
Si204	Si	0.33606	0.73414	0.65393
Si205	Si	0.46405	0.64891	0.65350
Si206	Si	0.33894	0.89878	0.65398
O207	O	0.43205	0.48725	0.56627
O208	O	0.30706	0.73672	0.56641
O209	O	0.44784	0.64273	0.56591
O210	O	0.32330	0.89249	0.56648
O211	O	0.32347	0.58076	0.56734
O212	O	0.44865	0.83029	0.56776
O213	O	0.29528	0.67192	0.68999
O214	O	0.42019	0.92172	0.68981
O215	O	0.45214	0.56782	0.69018
O216	O	0.32604	0.81733	0.68922
O217	O	0.41799	0.71479	0.67807
O218	O	0.29263	0.96461	0.67797
Al219	Al	0.38745	0.90276	0.51008
Al220	Al	0.26202	0.65295	0.50968
Si221	Si	0.31311	0.48844	0.36533
Si222	Si	0.43792	0.73791	0.36570
Si223	Si	0.30997	0.82353	0.36606
Si224	Si	0.43462	0.57359	0.36507
O225	O	0.34199	0.98578	0.45362
O226	O	0.46650	0.73604	0.45309
O227	O	0.32610	0.82978	0.45349
O228	O	0.45034	0.58000	0.45291
O229	O	0.45061	0.89229	0.45193
O230	O	0.32546	0.64212	0.45206
O231	O	0.47835	0.80132	0.33054
O232	O	0.35356	0.55035	0.32871
O233	O	0.32287	0.90501	0.33071
O234	O	0.44798	0.65509	0.33008

O235	O	0.35618	0.75820	0.34118
O236	O	0.48109	0.50745	0.34148
H237	H	0.30821	0.53994	0.58674
H238	H	0.43280	0.78899	0.58608
H239	H	0.46763	0.93362	0.43482
H240	H	0.34144	0.68244	0.43271
Al241	Al	0.63672	0.56978	0.50943
Al242	Al	0.76163	0.82021	0.50888
Si243	Si	0.71114	0.98491	0.65396
Si244	Si	0.58587	0.73433	0.65311
Si245	Si	0.71424	0.64908	0.65331
Si246	Si	0.58951	0.89896	0.65380
O247	O	0.68237	0.48674	0.56616
O248	O	0.55738	0.73658	0.56582
O249	O	0.69795	0.64306	0.56583
O250	O	0.57316	0.89257	0.56627
O251	O	0.57320	0.58047	0.56726
O252	O	0.69858	0.83095	0.56738
O253	O	0.54520	0.67206	0.68899
O254	O	0.67088	0.92160	0.68989
O255	O	0.70135	0.56777	0.68895
O256	O	0.57633	0.81741	0.68872
O257	O	0.66763	0.71467	0.67735
O258	O	0.54300	0.96431	0.67890
Al259	Al	0.63712	0.90328	0.50948
Al260	Al	0.51195	0.65283	0.50926
Si261	Si	0.56295	0.48823	0.36527
Si262	Si	0.68775	0.73826	0.36487
Si263	Si	0.55994	0.82377	0.36583
Si264	Si	0.68508	0.57373	0.36537
O265	O	0.59113	0.98624	0.45313
O266	O	0.71615	0.73616	0.45258
O267	O	0.57604	0.82982	0.45331
O268	O	0.70073	0.58002	0.45290
O269	O	0.70036	0.89291	0.45155
O270	O	0.57531	0.64200	0.45146
O271	O	0.72818	0.80149	0.32949
O272	O	0.60374	0.55099	0.32990
O273	O	0.57270	0.90490	0.33032
O274	O	0.69774	0.65502	0.32975
O275	O	0.60574	0.75741	0.34174
O276	O	0.73088	0.50740	0.34118
H277	H	0.55584	0.53940	0.58429
H278	H	0.68285	0.79013	0.58621
H279	H	0.71689	0.93519	0.43536
H280	H	0.59018	0.68187	0.43035
Mg281	Mg	0.88654	0.56994	0.50938
Al282	Al	1.01154	0.82003	0.50875
Si283	Si	0.96065	0.98457	0.65248
Si284	Si	0.83610	0.73481	0.65305
Si285	Si	0.96408	0.64918	0.65337
Si286	Si	0.83906	0.89961	0.65256
O287	O	0.93201	0.48711	0.56607
O288	O	0.80685	0.73708	0.56570
O289	O	0.94784	0.64282	0.56571

O290	O	0.82312	0.89271	0.56540
O291	O	0.82330	0.58089	0.56710
O292	O	0.94834	0.83085	0.56684
O293	O	0.79515	0.67298	0.68955
O294	O	0.92023	0.92196	0.68855
O295	O	0.95124	0.56795	0.68947
O296	O	0.82578	0.81821	0.68819
O297	O	0.91790	0.71530	0.67747
O298	O	0.79289	0.96490	0.67773
Al299	Al	0.88704	0.90318	0.50866
Al300	Al	0.76196	0.65317	0.50904
Si301	Si	0.81281	0.48814	0.36496
Si302	Si	0.93749	0.73802	0.36469
Si303	Si	0.80961	0.82405	0.36489
Si304	Si	0.93445	0.57350	0.36493
O305	O	0.84166	0.98624	0.45244
O306	O	0.96655	0.73610	0.45226
O307	O	0.82584	0.83000	0.45241
O308	O	0.95046	0.57982	0.45276
O309	O	0.95053	0.89255	0.45094
O310	O	0.82557	0.64207	0.45175
O311	O	0.97853	0.80057	0.32899
O312	O	0.85354	0.55003	0.32849
O313	O	0.82277	0.90534	0.32956
O314	O	0.94805	0.65479	0.32976
O315	O	0.85569	0.75786	0.34070
O316	O	0.98123	0.50756	0.34124
H317	H	0.80724	0.53931	0.58447
H318	H	0.93206	0.78954	0.58455
H319	H	0.96678	0.93491	0.43472
H320	H	0.84115	0.68218	0.43163
C321	C	0.48928	0.91565	0.17338
H322	H	0.48404	0.97611	0.17896
C323	C	0.47665	0.79169	0.17465
C324	C	0.46255	0.71586	0.17782
H325	H	0.41651	0.69654	0.19041
C326	C	0.50973	0.66414	0.16779
C327	C	0.56887	0.69306	0.15409
H328	H	0.61005	0.65691	0.14865
N329	N	0.58173	0.76709	0.14754
C330	C	0.53517	0.81094	0.15934
C331	C	0.38329	0.87911	0.20291
H332	H	0.39489	0.91246	0.25337
H333	H	0.35578	0.82888	0.21155
N334	N	0.33480	0.92541	0.14582
C335	C	0.27434	0.94854	0.17453
H336	H	0.29497	0.98423	0.22224
H337	H	0.25325	0.89717	0.19102
C338	C	0.22213	0.98941	0.11630
H339	H	0.24257	1.04387	0.10587
H340	H	0.17868	1.00173	0.13757
C341	C	0.20033	0.94292	0.04626
H342	H	0.16255	0.97391	0.00533
H343	H	0.17511	0.89112	0.05605
C344	C	0.26139	0.92365	0.01835

H345	H	0.24704	0.88902	-0.03125
H346	H	0.28352	0.97547	0.00418
C347	C	0.31450	0.88061	0.07372
H348	H	0.29562	0.82568	0.08476
H349	H	0.36065	0.87159	0.05658
C350	C	0.60386	0.93009	0.14418
H351	H	0.63121	0.89230	0.11678
H352	H	0.63898	0.94299	0.19728
C353	C	0.58560	1.00106	0.10123
C354	C	0.57326	1.06763	0.13406
H355	H	0.57770	1.06869	0.19234
C356	C	0.55502	1.13268	0.09468
H357	H	0.54468	1.18409	0.12167
C358	C	0.54927	1.13234	0.02074
H359	H	0.53378	1.18293	-0.01120
C360	C	0.56305	1.06671	-0.01255
H361	H	0.55832	1.06556	-0.07087
C362	C	0.58112	1.00213	0.02764
H363	H	0.59087	0.95078	0.00013
N364	N	0.54613	0.88827	0.15670
H365	H	0.35976	0.97215	0.13860
C366	C	0.44674	0.85992	0.18461
C367	C	0.54338	0.53631	0.15908
H368	H	0.52674	0.47963	0.16778
H369	H	0.54731	0.53979	0.10344
H370	H	0.59322	0.54617	0.19692
O371	O	0.49565	0.58871	0.17109
C372	C	0.19141	0.59055	0.16859
H373	H	0.15321	0.59646	0.19809
C374	C	0.26515	0.54813	0.11183
C375	C	0.30830	0.51412	0.07683
H376	H	0.31483	0.45315	0.07723
C377	C	0.34357	0.55910	0.04083
C378	C	0.33327	0.63736	0.04285
H379	H	0.35930	0.67691	0.01642
N380	N	0.29114	0.67157	0.07691
C381	C	0.26034	0.62415	0.10876
C382	C	0.19935	0.45046	0.17075
H383	H	0.22061	0.44152	0.22901
H384	H	0.21933	0.40543	0.14310
N385	N	0.12357	0.43637	0.15515
C386	C	0.11279	0.36067	0.18934
H387	H	0.13561	0.36730	0.24774
H388	H	0.14127	0.31792	0.16838
C389	C	0.03801	0.34096	0.16985
H390	H	0.01097	0.38115	0.19557
H391	H	0.03253	0.28582	0.19281
C392	C	0.00667	0.34012	0.08789
H393	H	-0.04739	0.32564	0.07514
H394	H	0.03017	0.29604	0.06238
C395	C	0.01592	0.41646	0.05625
H396	H	-0.00477	0.41513	-0.00287
H397	H	-0.01250	0.45952	0.07694
C398	C	0.08982	0.43931	0.07280
H399	H	0.11774	0.40067	0.04664

H400	H	0.09644	0.49667	0.05556
C401	C	0.18922	0.73198	0.14287
H402	H	0.21761	0.76848	0.11615
H403	H	0.20027	0.75236	0.19894
C404	C	0.11491	0.73739	0.10556
C405	C	0.08920	0.71692	0.03295
H406	H	0.12376	0.69789	0.00198
C407	C	0.02040	0.71824	-0.00057
H408	H	0.00070	0.69979	-0.05709
C409	C	-0.02419	0.74125	0.03829
H410	H	-0.07866	0.74182	0.01241
C411	C	0.00088	0.76292	0.11058
H412	H	-0.03383	0.78054	0.14196
C413	C	0.06971	0.76079	0.14346
H414	H	0.08831	0.77680	0.20077
N415	N	0.21509	0.65482	0.14381
H416	H	0.10398	0.47754	0.17980
C417	C	0.21981	0.52554	0.15056
C418	C	0.42028	0.57006	-0.03318
H419	H	0.45186	0.53414	-0.05719
H420	H	0.38402	0.59913	-0.07686
H421	H	0.45296	0.61128	0.00279
O422	O	0.38613	0.52473	0.00587
C423	C	0.59297	0.46241	0.84527
H424	H	0.54094	0.48110	0.83588
C425	C	0.70342	0.45425	0.85858
C426	C	0.77205	0.45761	0.86478
H427	H	0.79616	0.51089	0.85840
C428	C	0.80884	0.39158	0.87699
C429	C	0.77375	0.32541	0.88508
H430	H	0.79875	0.27099	0.89570
N431	N	0.70682	0.32192	0.88080
C432	C	0.67648	0.38646	0.86689
C433	C	0.64668	0.58632	0.82955
H434	H	0.61584	0.59670	0.77421
H435	H	0.69733	0.60775	0.83278
N436	N	0.61859	0.63577	0.87942
C437	C	0.62260	0.71902	0.85864
H438	H	0.59239	0.72416	0.80248
H439	H	0.67564	0.73149	0.86168
C440	C	0.59703	0.76904	0.91008
H441	H	0.54286	0.75905	0.90261
H442	H	0.60225	0.82758	0.89530
C443	C	0.63620	0.75607	0.98877
H444	H	0.61700	0.79244	1.02535
H445	H	0.68942	0.77225	0.99679
C446	C	0.63156	0.67371	1.00823
H447	H	0.66177	0.66360	1.06380
H448	H	0.57908	0.65900	1.00566
C449	C	0.65761	0.62189	0.95881
H450	H	0.71115	0.63283	0.96367
H451	H	0.65119	0.56248	0.96983
C452	C	0.56039	0.32543	0.86386
H453	H	0.58880	0.27261	0.87346
H454	H	0.52430	0.31843	0.81040

C455	C	0.52365	0.33687	0.92136
C456	C	0.55901	0.34639	0.99384
H457	H	0.61427	0.34672	1.00845
C458	C	0.52596	0.35567	1.04740
H459	H	0.55506	0.36258	1.10413
C460	C	0.45572	0.35608	1.02889
H461	H	0.42922	0.36376	1.07076
C462	C	0.41956	0.34754	0.95652
H463	H	0.36421	0.34827	0.94094
C464	C	0.45351	0.33779	0.90374
H465	H	0.42436	0.33072	0.84699
N466	N	0.60697	0.38725	0.85930
H467	H	0.56918	0.62208	0.87164
C468	C	0.64920	0.50434	0.84400
C469	C	0.90504	0.32958	0.85837
H470	H	0.94431	0.34663	0.83294
H471	H	0.92758	0.29418	0.90473
H472	H	0.86710	0.29706	0.81811
O473	O	0.87692	0.39443	0.88191
C474	C	0.21187	0.10828	0.85584
H475	H	0.23097	0.05627	0.83871
C476	C	0.14734	0.20288	0.87270
C477	C	0.10096	0.25866	0.87524
H478	H	0.04657	0.24723	0.85759
C479	C	0.12485	0.33094	0.89538
C480	C	0.19555	0.34218	0.91511
H481	H	0.21873	0.39711	0.93226
N482	N	0.24132	0.28800	0.91349
C483	C	0.21371	0.22289	0.89089
C484	C	0.08863	0.07426	0.82035
H485	H	0.08764	0.05670	0.76501
H486	H	0.04071	0.10321	0.81610
N487	N	0.08566	0.00191	0.86370
C488	C	0.13009	-0.05925	0.84264
H489	H	0.18281	-0.04167	0.86467
H490	H	0.11868	-0.05976	0.78384
C491	C	0.11656	-0.13653	0.86898
H492	H	0.13632	-0.14034	0.92785
H493	H	0.14499	-0.17737	0.84553
C494	C	0.04154	-0.15676	0.84808
H495	H	0.03408	-0.21286	0.86782
H496	H	0.02137	-0.16010	0.78879
C497	C	0.00283	-0.09765	0.87852
H498	H	-0.05134	-0.11158	0.86385
H499	H	0.02018	-0.09601	0.93791
C500	C	0.01047	-0.02130	0.84736
H501	H	-0.00983	-0.02256	0.78867
H502	H	-0.01454	0.02377	0.86901
C503	C	0.33108	0.16785	0.89088
H504	H	0.35437	0.20666	0.93552
H505	H	0.34024	0.19261	0.84233
C506	C	0.36392	0.09204	0.90477
C507	C	0.38792	0.06575	0.97489
H508	H	0.38587	0.10261	1.02034
C509	C	0.41642	-0.00486	0.98823

H510	H	0.43765	-0.02287	1.04389
C511	C	0.42125	-0.05118	0.93098
H512	H	0.44538	-0.10615	0.94106
C513	C	0.39764	-0.02546	0.86055
H514	H	0.40233	-0.06058	0.81495
C515	C	0.36943	0.04541	0.84803
H516	H	0.35157	0.06532	0.79224
N517	N	0.25743	0.16601	0.88181
H518	H	0.10327	0.01479	0.91662
C519	C	0.14569	0.12755	0.84999
C520	C	0.09581	0.45789	0.87208
H521	H	0.04930	0.49003	0.85161
H522	H	0.12983	0.48976	0.91646
H523	H	0.11967	0.45114	0.82774
O524	O	0.07951	0.38743	0.89688
C525	C	0.83996	0.99671	0.08876
H526	H	0.85603	1.05181	0.07586
C527	C	0.79432	0.90116	0.13244
C528	C	0.76520	0.84621	0.16504
H529	H	0.73441	0.86078	0.20160
C530	C	0.77765	0.77125	0.15277
C531	C	0.81874	0.75571	0.10633
H532	H	0.83090	0.69848	0.09300
N533	N	0.84751	0.80882	0.07412
C534	C	0.83373	0.87721	0.09021
C535	C	0.76548	1.04148	0.16349
H536	H	0.78391	1.03969	0.22243
H537	H	0.71067	1.03437	0.15059
N538	N	0.77815	1.12143	0.14260
C539	C	0.76112	1.17727	0.19755
H540	H	0.79367	1.16297	0.25112
H541	H	0.70830	1.16709	0.19700
C542	C	0.77182	1.25704	0.17537
H543	H	0.82580	1.26714	0.18148
H544	H	0.75701	1.29615	0.21290
C545	C	0.73091	1.27245	0.09761
H546	H	0.73940	1.33048	0.08310
H547	H	0.67677	1.26767	0.09406
C548	C	0.74868	1.21689	0.04438
H549	H	0.71736	1.22647	-0.01109
H550	H	0.80149	1.22459	0.04367
C551	C	0.73693	1.13673	0.06395
H552	H	0.68377	1.12740	0.06161
H553	H	0.75136	1.09518	0.02840
C554	C	0.90834	0.93074	0.01208
H555	H	0.89634	0.87927	-0.02099
H556	H	0.96063	0.92436	0.04509
C557	C	0.90190	0.99804	-0.03593
C558	C	0.85545	0.99810	-0.10377
H559	H	0.82364	0.94841	-0.12243
C560	C	0.84847	1.05983	-0.14834
H561	H	0.81210	1.05853	-0.20203
C562	C	0.88775	1.12370	-0.12508
H563	H	0.88192	1.17165	-0.16125
C564	C	0.93371	1.12510	-0.05674

H565	H	0.96430	1.17534	-0.03736
C566	C	0.94059	1.06247	-0.01313
H567	H	0.97640	1.06392	0.04091
N568	N	0.86415	0.93438	0.06095
H569	H	0.82824	1.12670	0.14689
C570	C	0.79713	0.97997	0.13068
C571	C	0.77943	0.64501	0.19237
H572	H	0.75923	0.61151	0.22920
H573	H	0.76866	0.61569	0.13995
H574	H	0.83426	0.65004	0.21517
O575	O	0.74863	0.71665	0.18506
O576	O	0.80869	0.68306	0.85265
H577	H	0.82558	0.66254	0.81347
H578	H	0.79475	0.73345	0.83451
O579	O	0.91615	0.14241	0.18417
H580	H	0.93003	0.15289	0.23644
H581	H	0.95426	0.11115	0.18037
O582	O	0.63906	0.09354	0.79441
H583	H	0.66616	0.08516	0.76031
H584	H	0.60539	0.12801	0.76643
O585	O	0.75997	0.82448	0.78993
H586	H	0.72398	0.86046	0.77105
H587	H	0.78380	0.82675	0.75234
O588	O	0.94633	0.54368	0.93021
H589	H	0.93018	0.56585	0.88202
H590	H	0.92657	0.49396	0.91838
O591	O	0.88027	0.56132	0.78287
H592	H	0.84667	0.52858	0.75075
H593	H	0.90894	0.57099	0.75058
O594	O	0.48060	0.61880	0.82568
H595	H	0.44220	0.65255	0.81532
H596	H	0.46755	0.58696	0.78193
O597	O	0.83601	0.60315	0.98333
H598	H	0.82671	0.63676	0.94173
H599	H	0.88123	0.58663	0.98341
O600	O	0.59822	0.95565	0.83558
H601	H	0.56858	0.95533	0.78664
H602	H	0.62013	1.00457	0.83538
O603	O	0.02614	0.04161	0.19230
H604	H	0.01132	-0.00782	0.20287
H605	H	0.05828	0.05412	0.23900
